# Insulin resistance, metabolic phenotypes, and the obesity paradox: a triglyceride glucose-body mass index framework in partial kidney diseases

**DOI:** 10.3389/fendo.2026.1915011

**Published:** 2026-09-23

**Authors:** Xiangzhe Sun, Lu Bai, Changchang Liang, Shuai Guo, Yongliang Chen, Maodong Liu

**Affiliations:** Department of Nephrology, Hebei Medical University Third Hospital, Shijiazhuang, China

**Keywords:** CKM syndrome, insulin resistance, kidney disease, obesity paradox, TyG-BMI

## Abstract

**Background:**

Insulin resistance(IR) is a key driver of cardiorenal-metabolic diseases. The Triglyceride Glucose-Body Mass Index (TyG-BMI), a composite marker of lipid-glucose metabolism and overall adiposity, has gained traction as a predictive tool. Its clinical application, however, is complicated by non-linear and sometimes paradoxical associations with clinical outcomes, a phenomenon reminiscent of the”obesity paradox”. This review critically evaluates the performance of TyG-BMI across the spectrum of kidney disease, aiming to reconcile these divergent findings through a metabolic-endocrine framework.

**Methods:**

We performed a narrative synthesis of observational studies and secondary analyses of large-scale public databases such as National Health and Nutrition Examination Survey(NHANES), China Health and Retirement Longitudinal Study(CHARLS), UK Biobank, Medical Information Mart for Intensive Care IV(MIMIC-IV), identified through PubMed and Google Scholar, to evaluate the association of TyG-BMI with the incidence, progression, and prognosis of acute kidney injury (AKI), diabetic kidney disease (DKD), chronic kidney disease (CKD), and Cardiovascular-Kidney-Metabolic syndrome(CKM).

**Results:**

TyG-BMI shows significant, context-dependent predictive value. In early-stage or metabolically heterogeneous populations, elevated TyG-BMI consistently predicts higher disease risk. Conversely, in advanced stages (e.g., end-stage renal disease, stage 4 CKM), the association frequently becomes U-shaped, with low TyG-BMI paradoxically linked to excess mortality. This reflects its dual nature: the TyG component captures ‘metabolic toxicity’, while BMI proxies ‘nutritional reserve’. Thus, the ‘obesity paradox’ may represent a metabolic phenotype spectrum—from metabolically healthy obesity (MHO) to metabolically unhealthy lean (MUL)—that the combination of its TyG and BMI components helps differentiate. For disease incidence prediction, TyG-BMI is often outperformed by indices of central obesity (e.g., Triglyceride-Glucose-Waist Circumference Index(TyG-WC), Triglyceride-Glucose–Waist-to-Height Ratio(TyG-WHtR); for mortality in advanced illness, the standalone TyG index may be superior.

**Conclusion:**

TyG-BMI is a valuable tool for metabolic phenotyping and risk stratification in kidney disease, with its paradoxical associations highlighting the complex interplay between metabolic health and nutritional reserve. Future research must establish context-specific clinical thresholds. TyG-BMI is preferentially useful in settings where waist circumference is unavailable, in early CKD screening, and in select type 2 diabetes mellitus (T2DM) subgroups. In advanced CKD, dialysis, critical illness, and CKM stage 4, alternative indices such as TyG index, TyG-WC, or TyG-WHtR should be prioritized due to the confounding effects of fluid overload and catabolic state.

## 1 Background

At the World Health Assembly(WHA) convened on May 23, 2025, the first-ever WHA resolution on kidney health was adopted, recognizing kidney disease as a growing global public health issue and urging Member States to promote it as a noncommunicable disease of growing global priority. This landmark resolution—the result of years of global advocacy—formally integrates chronic kidney disease(CKD) into the WHO Global Noncommunicable Diseases agenda. CKD currently affects approximately 850 million people worldwide and is projected to become the fifth leading cause of death by 2040, highlighting the massive global burden of kidney disease and the anticipated need for additional resource allocation to address the challenges it poses.This accomplishment formally acknowledges the massive global burden of kidney disease and is expected to encourage the distribution of additional funds to address the grave issues it poses to humanity (1). Kidney disease development and progression are significantly influenced by insulin resistance(IR).The Triglyceride Glucose-Body Mass Index (TyG-BMI) has been found to be a non-insulin indicator of Insulin resistance(IR) (2). Numerous studies have now demonstrated the significant impact that TyG-BMI plays in digestive and cardiovascular disorders. This article primarily summarizes the current research status and contradictions regarding TyG-BMI in kidney diseases.

Given the breadth and rapidly evolving nature of the topic, we conducted a narrative synthesis rather than a formal systematic review. Literature searches were performed primarily in Google Scholar (up to December 2025, with an update in March 2026 to incorporate the most recent publications) using combinations of the following keywords: “TyG-BMI”, “kidney disease”, “chronic kidney disease(CKD)”, “diabetic kidney disease(DKD)”, “acute kidney injury(AKI)”, “obesity paradox”, “cardio-renal-metabolic multimorbidity(CRMM)”, and “Cardiovascular-kidney-metabolic (CKM) syndrome”. Search terms were entered individually and in combination (using AND/OR logic) to maximize coverage. PubMed was also consulted for cross-validation. Reference lists of key articles were manually screened to identify additional relevant studies.

The nomenclature describing the interplay among cardiovascular, kidney, and metabolic diseases has evolved. Earlier literature commonly used the term “cardio-renal-metabolic multimorbidity (CRMM)”. In 2023, the American Heart Association (AHA) formally introduced the broader and more structured concept of “Cardiovascular-Kidney-Metabolic (CKM) syndrome”, encompassing a staging system (Stages 0–4). This review respects this chronological evolution: for studies published before 2023, we retain the original term “CRMM”; when referring to the integrated syndrome and its staging framework from 2023 onward, we adopt “CKM”. This distinction clarifies the historical context and conceptual framework of the evidence.

Inclusion criteria for the final selection of 46 cited works were: ① studies published between 2020 and 2026; ② original research (observational studies, cohort studies, cross-sectional analyses) or authoritative reviews/guidelines; ③ studies reporting associations between TyG-BMI or related indices and kidney disease outcomes (incidence, progression, mortality); ④ studies providing mechanistic insights, large-scale epidemiological data, or conflicting findings that inform the discussion of the obesity paradox. Exclusion criteria​were: ① case reports, editorials, commentaries without original data; ② animal or cell-based studies; ③ duplicate publications from the same dataset (the most complete version was retained).

Because this is a narrative review designed to synthesize conceptual frameworks rather than to quantify pooled estimates, a formal PRISMA flow diagram was not constructed. However, the search and selection process was iterative: initial broad searches yielded approximately 600–700 records; after deduplication and title/abstract screening, 46 were ultimately selected based on relevance, data richness, and contribution to the thematic framework. Background materials (e.g., WHO resolutions, GBD reports, guidelines) were retrieved separately via targeted hand-searching and are not part of the systematic synthesis.

As this is a narrative review aimed at synthesizing conceptual frameworks and mechanistic insights rather than deriving pooled quantitative estimates, we did not perform formal data extraction for meta-analysis. Instead, two reviewers (XS and LB) independently extracted thematic information from each of the 46 core analytical studies, including: study design, population characteristics (sample size, age range, geographic region), reported outcomes (incidence, prevalence, mortality, disease progression), key findings regarding TyG-BMI or related indices, and any reported nonlinear associations (e.g., U-shaped, J-shaped, L-shaped patterns). Discrepancies were resolved through discussion with a third reviewer (ML). This information was organized into thematic tables (**Supplementary Table 1**) to facilitate cross-study comparison.

Regarding study quality, we acknowledge that a formal risk-of-bias assessment (e.g., Newcastle-Ottawa Scale or JBI checklist) was not conducted. This decision was deliberate and appropriate for a narrative review for the following reasons: ① the included core studies span highly heterogeneous designs (prospective cohorts, retrospective cohorts, cross-sectional studies, *post-hoc* analyses of clinical trials), making a single quality instrument unsuitable; ② the primary objective is to synthesize conceptual and mechanistic evidence, not to generate weighted effect estimates; ③the obesity paradox literature itself is characterized by diverse methodologies, and imposing a uniform quality threshold could inadvertently exclude valuable hypothesis-generating evidence. Instead, we adopted a narrative evidence-strength assessment by categorizing studies according to design hierarchy (large prospective cohorts > smaller cohorts > cross-sectional studies) and documenting potential biases (confounding, selection bias, measurement error) in a dedicated section (Section 4.6: Methodological Limitations of Current Evidence). Readers are encouraged to consult the original publications for detailed methodological reporting.

## 2 TyG-BMI overview

We propose that the development of TyG-BMI can be conceptually divided into three phases.The discovery of the glucose clamp technique in 1966 established the gold standard for assessing insulin secretion and resistance in the first phase, but due to its complexity, it cannot be widely applied in clinics.Because of this limitation, there is an urgent need for simple and reliable alternative indicators. After that, models like Homeostatic Model Assessment for Insulin Resistance(HOMA-IR) that use fasting insulin and blood glucose have been used a lot, which has helped with some real-world problems (3). But there are still problems with insulin detection, such as a lack of standardization and high costs. To tackle this issue, researchers have investigated additional indicators that do not depend on insulin detection, leading to the emergence of the TyG-BMI index as necessitated by current circumstances. It allows for the screening of IR using data from regular physical exams, which is a new step in making IR easier to measure.Fernando Guerrero Romero introduced the Triglyceride Glucose (TyG) index in 2010,the formula is ln[fasting triglycerides (mg/dL) × fasting blood glucose (mg/dL)/2], and it has a high level of specificity and sensitivity for detecting IR. One non-insulin-based measure of IR is the TyG index (4). In the second phase, body mass index (BMI), fasting blood glucose (FBG), and fasting triglycerides (TG) are combined to create the TyG-BMI composite index. Its definition is ln[fasting TG (mg/dL) × FBG (mg/dL)/2] × BMI (2). The core strength of TyG-BMI lies in its dual capability to simultaneously reflect glucose-lipid metabolic disorders (via TyG components) and obesity severity (via BMI components). This characteristic enables TyG-BMI to function across a wide variety of applications. In the third phase, TyG-BMI has expanded to include additional non-insulin-based indicators of IR, such as Triglyceride-Glucose-Waist Circumference Index (TyG-WC) and Triglyceride-Glucose–Waist-to-Height Ratio(TyG-WHtR), broadening the TyG index and its related indices clinical utility.

TyG-BMI demonstrates three key predictive values: Predicting risk, illness progression, and an adverse outcome. The risk prediction value assesses whether high-risk individuals will progress to disease stages. The progression prediction value assesses whether patients in specific disease stages will maintain stability or experience disease progression. The adverse prognosis prediction value quantifies the risk of adverse events following disease progression. In addition, the predictive role of TyG-BMI isn’t exclusive to kidney illness in one system. but also shows increasing significance in kidney-related multisystem diseases such as CKM.

Even though TyG-BMI has demonstrated promise in a number of kidney disorders,TyG-BMI still faces significant clinical complexities. Its ability to predict various clinical situations and prognosis outcomes varies, frequently exhibiting inverse or U-shaped relationships—a phenomenon akin to the “obesity paradox” (5, 6). This review will leverage the conceptual framework of the obesity paradox to reconcile these heterogeneous findings and elucidate the underlying mechanisms.

## 3 TyG-BMI and the obesity paradox: a phenotypic framework

The frequent U-shaped, J-shaped, or L-shaped relationships between TyG-BMI and clinical outcomes in kidney diseases may not be purely statistical artifacts; instead, they likely reflect a pathophysiological complexity that refines the concept of the “obesity paradox”. It should be acknowledged, however, that this interpretation is based on observational and database-derived evidence, and residual confounding or reverse causality cannot be excluded. This section establishes a phenotypic framework—grounded in the distinction between metabolic health and nutritional status—that will guide the interpretation of clinical evidence in subsequent sections.

### 3.1 The obesity paradox: beyond BMI

The obesity paradox refers to the seemingly counterintuitive observation that overweight or mildly obese individuals with established cardiovascular or renal disease may have better prognoses than their normal-weight counterparts. Traditional measures like BMI are inadequate to explain this phenomenon because they conflate fat mass with lean mass and cannot distinguish between “metabolically healthy” and “metabolically unhealthy” obesity.

### 3.2 TyG-BMI as a tool for metabolic phenotyping

#### 3.2.1 Four-quadrant model​

To apply the conceptual framework of the “obesity paradox” in explaining the nonlinear correlations (U-shaped, J-shaped, or L-shaped) of TyG-BMI across different stages of kidney disease, we introduce a four-quadrant metabolic phenotype classification model based on two independent dimensions: TyG and BMI. This model is not a direct output of the TyG-BMI index itself, but rather a heuristic analytical tool that helps us understand why the relative contributions of the “metabolic toxicity signal” (TyG) and the “nutritional reserve signal” (BMI) within TyG-BMI change dynamically at different disease stages (7):

High TyG/high BMI (metabolically unhealthy obesity, MUO): Represents the highest levels of metabolic toxicity and fat load, primarily driving disease onset and progression.

High TyG/low BMI (metabolically unhealthy lean, MUL): Also referred to as the “toxic lean type,” reflects increased catabolic metabolism and protein-energy wasting (PEW), and is associated with abnormally high risk in advanced disease.

Low TyG/high BMI (metabolically healthy obesity, MHO): Associated with lower-than-expected risk, and is the main source of the obesity paradox observed in cross-sectional studies.

Low TyG/low BMI (metabolically healthy lean, MHL): Serves as the reference population with the lowest baseline risk.

#### 3.2.2 MUO: pathophysiology outline

The MUO (high TyG/high BMI) phenotype reflects visceral fat-driven insulin resistance, which selectively damages the glomerular endothelium through multiple pathways and constitutes the core initiating event of obesity-associated glomerulonephritis (ORG).

The massive overflow of free fatty acids (NEFA) and release of pro-inflammatory adipokines such as tumor necrosis factor-alpha(TNF-α), interleukin-6(IL-6),driven by visceral fat accumulation not only induces systemic IR, but also activates the renin–angiotensin–aldosterone system (RAAS) (8).

Hyperinsulinemia in the IR state promotes afferent arteriolar dilation, whereas overactivation of the RAAS leads to efferent arteriolar constriction.

Together, these effects markedly elevate the glomerular capillary hydrostatic pressure gradient, producing a sustained state of high pressure and hyperfiltration (9).

This hemodynamic overload directly injures the glomerular endothelium: high shear stress disrupts the endothelial glycocalyx and increases vascular permeability (8).

Concurrently, elevated NEFA and inflammatory factors exacerbate endothelial dysfunction by upregulating Intercellular Cell Adhesion Molecule-1(ICAM-1) and Vascular Cell Adhesion Molecule-1(VCAM-1) and promoting oxidative stress (10).

Locally activated angiotensin II(Ang-II) in the kidney then directly triggers endothelial cell apoptosis and upregulates transforming growth factor-β (TGF-β) expression, fueling the progression of glomerular microvascular sclerosis and fibrosis. In summary, visceral fat-driven IR establishes the pathological basis for the onset and progression of ORG through a cascade axis of metabolic inflammation, hemodynamic overload, and direct endothelial injury.

#### 3.2.3 MUL: pathophysiology outline

MUL (high TyG/low BMI) does not merely represent “malnutrition” but rather a metabolic crisis defined by the coexistence of IR and PEW. Its hallmark is the vicious cycle of muscle depletion (sarcopenia) and systemic inflammation, forming one of the most potent mortality risk clusters in advanced CKD. The mechanistic chain proceeds as follows.

##### 3.2.3.1 IR with excessive motor protein breakdown

The insulin resistance indicated by a high TyG index is amplified in the CKD milieu—a setting of uremic toxins, acidosis, and RAAS activation. Impairment of the insulin/Insulin-like Growth Factor 1(IGF-1) pathway reduces PI3K/Akt phosphorylation, thereby disinhibiting FoxO transcription factors, upregulating atrogin-1 and MuRF1, activating the ubiquitin–proteasome system (UPS), and directly driving muscle protein degradation (11, 12).

##### 3.2.3.2 Low BMI: a marker of PEW and muscle wasting

A low BMI (<23 kg/m²) is a core diagnostic criterion for PEW (13). In late-stage CKD, low BMI is often not solely due to inadequate caloric intake; rather, it reflects inflammation-driven metabolic depletion—a state in which muscle amino acid utilization efficiency declines, leading to substantial anabolic resistance (14).

##### 3.2.3.3 Positive feedback loop between inflammation and PEW

The intersection of IR and low BMI essentially represents a pathological manifestation of metabolic–immune cross-talk:

Inflammation drives muscle wasting: IL-6 and TNF-α activate the UPS and autophagy via the NF-κB pathway, accelerating muscle breakdown, and simultaneously induce myostatin expression to further inhibit muscle synthesis (11, 15).

Muscle wasting promotes inflammation: Amino acids released from muscle can support acute-phase protein synthesis, but sustained muscle loss depletes metabolic substrates and diminishes antioxidant capacity, thereby exacerbating oxidative stress and pro-inflammatory cytokine release (16).

##### 3.2.3.4 Reinterpretation of “reverse epidemiology”

The MUL phenotype explains why low BMI is associated with a poorer prognosis in dialysis patients—the “low weight” in these patients essentially reflects inflammatory depletion and cachexia, not a healthy lean physique.

Their mortality risk is significantly higher than that of obese patients with elevated BMI but without muscle wasting (14, 17).

MUL (high TyG/low BMI) reflects an IR-driven positive feedback loop between PEW and inflammation—IR activation of the UPS exacerbates muscle wasting, muscle loss further amplifies systemic inflammation, and the two synergistically accelerate the progression of cachexia and mortality risk in late-stage CKD patients.

#### 3.2.4 Limitations

However, it should be clearly noted that TyG-BMI, as a single multiplicative index, cannot directly distinguish these four phenotypes on its own. This is because the index is a composite variable, and similar TyG-BMI values may arise from entirely different combinations of the TyG component (reflecting metabolic health) and the BMI component (reflecting nutritional reserves). For example, a patient with high TyG and low BMI (i.e., metabolically unhealthy lean type, MUL) and a patient with low TyG and high BMI (i.e., metabolically healthy obese type, MHO) may have nearly identical TyG BMI values, yet their pathophysiological status and clinical prognosis are completely opposite.

Therefore, we should not rely solely on a single TyG-BMI value for evaluation. Instead, we must explore the respective roles of metabolic health and nutritional reserves in the development and progression of disease in patients. We also look forward to the emergence of more comprehensive assessment indicators in the future that can better reflect the diverse physical conditions of patients.

### 3.3 Empirical evidence in kidney disease

Studies looking at the obesity paradox in CKD and End-Stage Renal Disease(ESRD) support the applicability of this framework (18–20). Three independent investigations (2010, 2014, 2017) converged on a key insight: the “obesity paradox” in BMI arises largely because BMI cannot distinguish between fat and muscle mass. Patients with kidney disease often have reduced muscle mass, and those with high BMI but preserved muscle mass—potentially identifiable by a lower TyG-BMI—may indeed have better outcomes. Conversely, normal-weight individuals with occult metabolic dysfunction (high TyG-BMI) may carry elevated risk that BMI alone fails to capture.

### 3.4 The critical modifier: fitness and body composition

Beyond the MHO/MUO distinction, two additional factors modulate the obesity paradox. First, cardiorespiratory fitness is a potent effect modifier. Patients with high TyG-BMI who maintain their fitness may reduce their metabolic risk by having better mitochondrial and insulin sensitivity (7). Second, fat distribution matters: TyG-BMI combined with WC can differentiate between metabolically harmful visceral fat and potentially protective subcutaneous fat. This explains why indices incorporating central obesity (TyG-WC, TyG-WHtR) consistently outperform TyG-BMI in predicting disease incidence.

### 3.5 Clinical implications: from static weight to dynamic health

The obesity paradox thus reframes clinical priorities: the goal should not be weight loss per se, but improvement in metabolic-functional health. TyG-BMI’s theoretical ability to track responses to exercise or pharmacologic interventions such as Sodium-glucose cotransporter 2(SGLT2i) suggests it could serve as a dynamic monitoring tool for this paradigm shift, although this hypothesis requires prospective validation in interventional trials. Future research should establish TyG-BMI thresholds for different metabolic phenotypes and integrate direct measures of muscle mass and fitness to capture the complex interplay among fatness, fitness, and metabolic health.

### 3.6 Evidence hierarchy for mechanistic claims linking TyG-BMI to kidney outcomes

We summarize the evidence supporting each mechanistic step in **Table 1**, where the evidence is categorized into three levels.

**Table 1:** Evidence hierarchy table.

Mechanistic claim	Evidence tier	Directly measured in TyG-BMI kidney cohorts?	Source of supporting evidence
Visceral adiposity → IR → glomerular hypertension/podocyte injury	Tier 2 (inferred)	No	ORG experimental & clinical studies
Lipotoxicity (FFA/ox-LDL) → mesangial proliferation/fibrosis	Tier 2 (inferred)	No	ORG & diabetic nephropathy basic science
PEW → uremic inflammation (CRP, IL-6) → muscle catabolism	Tier 2 (inferred)	No	ISRNM PEW consensus; CKD sarcopenia cohorts
Low TyG-BMI ↔ higher mortality in advanced CKD/HD/PD	Tier 1 (observed)	Yes (association, not mechanism)	Multiple cohort studies cited in Sections 4.2–4.5
Four-phenotype decomposition improves risk prediction	Tier 3 (hypothesis)	No	Proposed framework (this review)
Integration of fitness/body composition reclassifies risk	Tier 3 (hypothesis)	No	Lavie et al.; speculative

Tier 1 = directly observed association in reviewed studies; Tier 2 = mechanistically plausible based on independent literature; Tier 3 = hypothesis requiring prospective validation.

IR, Insulin Resistance; FFA, Free Fatty Acid; CRP, C-Reactive Protein; IL-6, Interleukin-6; CKD, Chronic Kidney Disease; HD, Hemodialysis; PD, Peritoneal Dialysis; ORG, Obesity-Related Glomerulopathy; ISRNM, International Society of Renal Nutrition and Metabolism; PEW, Protein-Energy Wasting; LDL, Low Density Lipoprotein.

## 4 TyG-BMI and kidney diseases

TyG-BMI and the clinical outcomes of kidney disorders have a number of intricate interactions, such as U-, J-, or L-shaped relationships. The “obesity paradox” of TyG-BMI in renal disorders will be specifically examined here, along with the correlation between TyG-BMI and prognosis in a number of kidney disease types.

### 4.1 TyG-BMI and AKI

The illness known as AKI includes a wide range of pathophysiologic processes and aetiologies that result in impaired kidney function. AKI is very common. One in five hospitalized adult patients—roughly half of all adult patients receiving critical care—and one in four pediatric patients undergoing intensive care experience AKI in high-resource settings.Significant mortality and long-term negative consequences, such as cardiovascular problems, CKD, and ESRD, are linked to each episode of AKI (21). Early detection of AKI can significantly reduce the occurrence of severe consequences.TyG-BMI has emerged as a promising biomarker for AKI risk across diverse clinical settings.

#### 4.1.1 AKI in heart failure patients

A study involving 1,250 patients from the Medical Information Mart for Intensive Care IV(MIMIC-IV) database and 2,300 from eICU Collaborative Research Database(eICU-CRD) found that elevated TyG-BMI levels were associated with increased risks of AKI and other serious consequences in HF patients, including a greater prevalence of Renal Replacement Therapy(RRT) use and in-hospital mortality (22). This suggests that blood lipids, blood glucose, and obesity are important risk factors for patients in critical condition. Health education should be a priority during diagnosis and treatment. The incidence of unfavorable clinical outcomes can eventually be decreased by managing body weight and poor metabolic status.

#### 4.1.2 Sepsis-associated acute kidney injury

Separation associated acute kidney injury (SA-AKI) is a significant component of acute renal injury and a frequent adverse event in patients in critical care units. A 2025 study revealed the high incidence of SA-AKI when applying the 2023 updated definition.187,888 adult ICU patients were included, and SA-AKI was found in nearly half of sepsis patients and about 1 in 6 ICU admissions. 37.7% of SA-AKI patients had at least one Major Adverse Kidney Events(MAKE) by the time they were released from the hospital, and 1 in 4 of them passed away while in the hospital(MAKE = death, kidney replacement therapy, or reduced kidney function from baseline) (23).

In patients with SA-AKI, elevated TyG-BMI typically indicates increased SA-AKI risk, though the relationship may reverse under specific conditions. A study of 1,117 sepsis patients demonstrated that while TyG-BMI showed a positive correlation with AKI, restricted cubic spline (RCS) analysis revealed a nonlinear relationship. Furthermore, when combined with simple disease severity scores (e.g., SOFA, SIRS), The prediction capacity of TyG-BMI was significantly enhanced (24). Another study involving 3,349 patients reached a different conclusion: TyG-BMI showed an L-shaped nonlinear relationship with short-term mortality in SA-AKI patients, with a clear threshold of 261.7. As TyG-BMI rose below this threshold, mortality fell; above it, mortality stabilized (25). These results are consistent with the “obesity paradox” that was previously mentioned.

This L-shaped relationship in SA-AKI patients can be reinterpreted through the lens of metabolic phenotypes and physiological reserve. As proposed by Lavie et al. (7), in critically ill patients with severely compromised ‘fitness’, the body’s priority shifts to survival. A very low TyG-BMI likely signifies a ‘double jeopardy’ scenario: catastrophic metabolic derangement (high TyG) compounded by profound energy depletion (low BMI), leading to high mortality. Conversely, a higher BMI may provide essential metabolic reserves, echoing the ‘obesity paradox’ observed in other advanced chronic diseases. This suggests that in end-stage catabolic states, the nutritional signal in TyG-BMI temporarily outweighs the metabolic signal for prognosis.In addition, there might be additional influencing factors such muscle mass, visceral fat thickness, cardiorespiratory fitness, and other “hidden indicators” that could have an impact. These potential modifiers—including muscle mass, visceral adiposity, and cardiorespiratory fitness—are not captured by simple metabolic (TyG) or anthropometric (BMI) indices alone, yet they profoundly influence survival in critical illness. Developing composite metrics that integrate these “hidden indicators” is a priority for future research.

#### 4.1.3 Contrast-induced nephropathy

The predictive value of TyG-BMI extends to specific clinical scenarios, particularly in evaluating contrast-induced nephropathy (CIN), previously known as contrast-induced acute kidney injury (CI-AKI). CIN, an AKI brought on by iodinated contrast agents, frequently follows coronary angiography and percutaneous coronary intervention (PCI). This illness is a significant source of iatrogenic renal impairment and accounts for about 11% of hospital-acquired renal dysfunction cases (26). While serum creatinine is the diagnostic laboratory indicators recommended by multiple guidelines for CIN, its delayed nature and susceptibility to confounding factors result in 20% of patients failing to meet CIN diagnostic thresholds based on serum creatinine levels. New biomarkers with potential for prediction in CIN include TyG-BMI. In diabetic patients having coronary artery bypass grafting (CABG), higher preoperative TyG-BMI levels significantly increase the risk of postoperative AKI (27). Regardless of diabetes status, TyG-BMI has been shown to be a reliable independent marker of CIN in patients with ST-segment elevation myocardial infarction (STEMI) receiving PCI in vascular interventional therapy. Direct comparison studies suggest that this indicator may predict CIN with greater accuracy than the TyG index alone (28, 29).

TyG-BMI can predict AKI risk, according to research based on ICU databases like MIMIC-IV and eICU-CRD; However, there is a lot of clinical variability in these databases. For example, ICU treatment interventions like nutritional support and the use of vasoactive medications may have an impact on the U-shaped relationship between TyG-BMI and mortality in septic AKI patients. Furthermore, the mechanistic explanation of TyG-BMI across various AKI subtypes is limited because public databases frequently lack comprehensive etiological information about AKI (such as ischemic vs. nephrotoxic AKI). To elucidate the causal relationship, we need more thorough prospective studies.

### 4.2 TyG-BMI and DKD

Worldwide, DKD remains highly prevalent, poorly diagnosed, and lacks new biomarkers for early detection. A population-based study carried out in China found that 2.9% of rural residents had DKD (30). Kidney diseases and diabetic nephropathy caused by metabolic disorders have a significant influence on the economics and healthcare systems of less developed countries (31). As an indicator of IR, TyG-BMI demonstrates complexity in the context of DKD. The varying prediction ability under various circumstances reflects this complexity.

In studies utilizing IR indicators to predict clinical outcomes for DKD, TyG-BMI shown excellent predictive value for all-cause mortality; however, its influence was nonlinear and had a U-shaped connection (32). This relationship may be comparable to what we discovered in AKI, where patients with TyG-BMI in the lower range have insufficient energy reserves and are unable to withstand multiple blows after DKD progresses to the more severe ESRD; in the high stage, the body’s metabolic disorder is too severe, and the mortality rate increases significantly. The metabolic imbalance and energy reserves achieve a “balance” in the intermediate stage, which lowers the death rate.In the meantime, “hidden indicators” including muscle mass, visceral fat thickness, and cardiorespiratory fitness should also be considered.

The U-shaped relationship in DKD mortality elegantly illustrates the interplay between metabolic health and nutritional status captured by the TyG component and the BMI component respectively. The elevated risk at the high TyG-BMI extreme corresponds to the MUO phenotype (7), where the toxicity of IR drives poor outcomes. The increased risk at the low TyG-BMI extreme may identify a subgroup with MUL characteristics, often associated with advanced protein-energy wasting. The nadir of the curve might represent a precarious balance or even a cluster of patients with relatively preserved metabolic health (MHO). This aligns with the concept that the relationship between adiposity and prognosis is not linear but is profoundly modified by underlying metabolic health, a cornerstone of the contemporary understanding of the obesity paradox.

The TyG-BMI demonstrates significant complexity in predicting the risk of DKD, with conflicting findings across studies. TyG-BMI has been shown to have predictive potential in several studies.The study included 1080 individuals with type 2 diabetes mellitus (T2DM) and found that TyG-BMI corresponds with renal impairment and functions as an independent risk factor for albuminuria, a critical sign of DKD (33). An National Health and Nutrition Examination Survey(NHANES) study with 6,891 individuals revealed a nonlinear correlation between TyG-BMI and the incidence of DKD. After correcting the model, there is a substantial positive correlation between TyG-BMI and the probability of getting DKD.The risk of DKD rises dramatically if TyG-BMI above the 288.5 level (34).

Conversely, other studies have identified limitations in TyG-BMI’s predictive value for DKD. In individuals with T2DM who do not have non-alcoholic fatty liver disease (NAFLD), TyG-BMI is a unique risk factor for DKD. But this association is only seen in NAFLD-free patients and not in NAFLD patients, according to a study which included 425 patients with T2DM.The main rationale might be because NAFLD is also an IR-related disorder, and the co-occurrence of DKD and NAFLD can interfere with the IR-related biomarker TyG-BMI’s ability to operate (35). Meanwhile,TyG-BMI and the DKD were found to be linearly related in a study of 924 people with T2DM, with the risk of DKD rising when TyG-BMI surpassed 243. However, the prediction performance was unsatisfactory (36).Other research have shown similar results. In a retrospective research of 521 individuals who have T2DM evaluating the association between alternative insulin resistance indicators and DKD, TyG-BMI was discovered to be a separate risk factor for DKD; Nevertheless, out of the four different measures of IR, it performed the least well in terms of prediction (37).

In some cases, TyG-BMI even loses its value in predicting the occurrence of DKD.After model adjustment, the differences in TyG-BMI across groups were no longer statistically significant in a study comprising 1,026 T2DM patients for predicting DKD through IR markers. Furthermore, TyG-BMI showed a poorer predictive value in comparison to other recently created modified markers such as TG/HDL-C, TyG, and TyG-WHtR (38). This complexity may be partly explained by a cluster analysis study involving 567 diabetic patients. The study detected four subgroups of T2DM: severe insulin-deficient diabetes (SIDD, 24.46%), age-related diabetes (MARD, 30.86%), mild obesity-related diabetes (MOD, 25.57%), and severe insulin-resistant diabetes (SIRD, 20.11%).Interestingly, those in the SIRD group with the highest TyG-BMI had the highest risk of dyslipidemia and DKD; this attribute is not present in other types of DKD (39). The intricate pathophysiology of DKD may be connected to this circumstance.As a result, TyG-BMI may not be appropriate for all T2DM patients, and in certain situations, it may not be a reliable indicator of DKD risk.

TyG-BMI shows potential in type 2 diabetes-related diabetic kidney disease, but its association with DKD risk in Type 1 Diabetes Mellitus (T1DM) remains to be explored. TyG-BMI was not a significant indicator of DKD in a study looking at the relationship between TyG index and DKD risk in T1DM. Nevertheless, there was still a strong correlation between the TyG index and the risk of DKD (40).

As a highly homogenous metabolic disease, DKD modifies the relative weights of the two major signals: “metabolic health” and “nutritional status” due to its distinct pathophysiological background. This is reflected in the observed decline in predictive performance or loss of significance after adjustment of TyG-BMI in the cohort of DKD patients, rather than suggesting that the indicator is ineffective (**Table 2**).

**Table 2:** Sources of heterogeneity in TyG-BMI’s predictive performance in DKD.

Source of heterogeneity	Key evidence	Mechanism	Clinical implication
T2DM subtype	Strong association confined to SIRD subgroup (39)	Insulin-resistant phenotype drives DKD risk;signal diluted in other subtypes	TyG-BMI most useful for identifying high-risk SIRD patients;not a universal DKD marker
Comorbidity (NAFLD)	Association lost in patients with NAFLD (35)	Co-existing hepatic insulin resistance disrupts TyG-BMI signal	Interpret TyG-BMI with caution in T2DM patients with concurrent NAFLD
Disease stage	U-shaped relationship with mortality (32)	Low TyG-BMI in advanced DKD reflects protein-energy wasting,not metabolic health	TyG-BMI predicts mortality non-linearly;low values in ESRD signal poor nutritional status
Treatment effects	SGLT2i/Glucagon-like peptide-1 receptor agonist(GLP-1RA) alter weight and glucose (7)	TyG-BMI change may reflect treatment response,not disease progression	Longitudinal TyG-BMI trajectories require adjustment for medication use
Statistical over-adjustment	Significance lost after adjusting for hypertension,proteinuria (38)	TyG-BMI is a comprehensive risk marker;adjusting for mediators attenuates effect	Avoid over-adjustment in multivariable models;TyG-BMI captures upstream risk

This table synthesizes evidence from studies above to illustrate the factors contributing to the variable performance of TyG-BMI in DKD. The robust correlation is limited to the SIRD subgroup (39) indicates TyG-BMI is most relevant for the insulin-resistant phenotype. Co-existing NAFLD (35), advanced disease stage (32), treatment effects (7), and statistical over-adjustment (38) all may attenuate its signal. Understanding these sources of heterogeneity is essential for appropriate clinical application.

### 4.3 TyG-BMI and CKD

CKD is currently a significant global public health issue. According to the latest data of the global burden of disease study published in the Lancet, in 2023, about 788million adults worldwide had CKD, with an age standardized prevalence of 14.2%. Currently, CKD ranks as the ninth leading cause of death globally. It is worth noting that China has the largest number of patients with CKD, with a prevalence of 12.3% and a population of 152million. Despite China’s remarkable progress in reducing CKD mortality, the vast population base, the disease burden associated with aging, and the prevalence of major metabolic risk factors like hyperglycemia, hypertension, and high body mass index constitute the critical and challenging aspects of CKD prevention and control in the future (41). As the disease worsens, the majority of CKD patients have poor nutritional status when they reach the ESRD stage.The TyG-BMI model, which integrates BMI—a key indicator of nutritional status—with IR,demonstrates significant potential in predicting the onset, progression, and clinical outcomes of CKD.

TyG-BMI alone may not be a reliable indicator of the likelihood of developing CKD.TyG-BMI and estimated Glomerular Filtration Rate(eGFR) had a negative correlation, while serum creatinine had a positive correlation, according to a China Health and Retirement Longitudinal Study(CHARLS) investigation that predicted the likelihood of CKD.Serum creatinine and eGFR are two crucial CKD diagnostic indicators.This suggests that TyG-BMI holds great potential for early CKD screening and treatment, which warrants further research (42). According to a prospective study with 3,485 healthy subjects, elevated TyG-BMI is a unique risk factor for the development of CKD and its measurement helps identify populations at greater risk early (43). TyG-BMI is not only a cross-sectional predictor in CKD risk prediction. A CHARLS study revealed an independent correlation between the likelihood of developing CKD and the cumulative average TyG-BMI (44), demonstrating that TyG-BMI has the ability to predict CKD risk both in the short and long term.

However,TyG-BMI and CKD had a nonlinear association, according to an NHANES study: while TyG-BMI showed a positive correlation with CKD, its predictive power was inferior to TyG-WHtR and TyG-WC, particularly in the U.S. population (45). Another NHANES study revealed a U-shaped relationship between TyG-BMI and CKD prevalence. TyG-BMI and CKD have a substantial nonlinear (U-shaped) connection, according to the RCS analysis, with a notable increase in CKD risk when TyG-BMI≥245 (6). This forecasting ability is negligible, though.This result confirms the nonlinear link between TyG-BMI and illness development, which is consistent with our previous findings in AKI and DKD, perhaps connected to the ‘obesity paradox’. Both studies found that TyG-BMI showed a positive correlation with CKD after adjusting the prediction model, though its risk assessment capability was inferior to TyG-WHtR. Future studies need to identify more and better new indicators.This suggests that when it comes to representing human body composition, BMI is less accurate than waist circumference(WC) and waist to height ratio(WHtR). WC and WHtR have greater predictive potential and can more accurately depict fat distribution, which in turn can be used as an indicator of visceral fat thickness.However, this circumstance might also be connected to the sources of the data. CHARLS originates from the Chinese population, while NHANES originates from the American population. One of the causes of the various effects of TyG-BMI is the objective variations in ethnicity.

TyG-BMI has limitations when it comes to CKD diagnosis. TyG-BMI demonstrated a substantial correlation with CKD in unadjusted models but lost significance following full correction in a cross-sectional study of a Chinese coastal community (46), suggesting that other metabolic and renal function parameters have a major impact on TyG-BMI ‘s capacity to predict CKD.Another disadvantage of BMI as a component is that complex comorbidities and low nutritional status interfere with the straightforward interpretation of BMI in cross-sectional research.In contrast, it is more beneficial to combine WC or WHtR indications. Our earlier analysis of the predictive power of TyG-BMI and CKD risk reflected this.This implies that TyG-BMI should be used in conjunction with other risk variables for a thorough assessment since it is not a reliable indicator of CKD on its own.

TyG-BMI is also useful for tracking CKD patients’ prognosis. A study with 3,089 participants revealed a non-linear U-shaped connection between TyG-BMI and cardiovascular and all-cause mortality in CKD patients (47). This non-linear association was also observed in AKI, which further prompts us to consider the ‘obesity paradox’ theory. A study on patients with stage 1–4 CKD demonstrated that the obesity paradox alters the predictive value of IR indices, with worse clinical outcomes being correlated with lower TyG-BMI levels. This finding necessitates more thorough research to clarify each predictive importance (48).

The TyG-BMI not only plays an important role in CKD, but also in Multiple Chronic Diseases (MCD) including CKD, reflecting the rise of multisystem diseases as a current trend. Two CHARLS based studies revealed an association between TyG-BMI and MCD, containing CKD, showing a positive correlation between TyG-BMI and increased risk of MCD. By monitoring this index, MCD can be minimized and controlled (49, 50). In this review, CKD denotes a single-organ disease characterized by a sustained decline in kidney function, and its diagnosis relies on indicators such as eGFR and proteinuria. Multiple chronic diseases (MCD) refers to a broader concept of comorbidity, denoting the coexistence of two or more chronic diseases in one individual, such as CKD with diabetes, hypertension, or coronary heart disease. MCD emphasizes the accumulation of multiple chronic conditions and their composite impact on overall health, rather than the pathological mechanisms of specific organ systems. The MCD literature reviewed here consists exclusively of studies involving CKD.

MCD and CKM are related yet distinct: MCD encompasses any combination of chronic diseases, while CKM defines a specific comorbidity pattern driven by metabolic abnormalities, with the cardiovascular, renal, and metabolic systems at its core. Therefore, in this article, when we cite studies related to MCD (such as references 49 and 50), we refer to a broad range of coexisting chronic diseases with diverse risk factors; CKM, on the other hand, has a clearer pathophysiological pathway and staging system. This distinction allows readers to accurately interpret the predictive value of TyG-BMI in different comorbidity.

The association between TyG-BMI and clinical outcomes in CKD study frequently shows a complicated nonlinear pattern, including a characteristic U-shaped relationship and oscillations or even elimination of predictive value. These events are not mutually exclusive; rather, they vividly illustrate the dynamic interplay between the “obesity paradox” notion and pathophysiological alterations in the course of CKD (**Table 3**).

**Table 3:** Stage-dependent performance and interpretation of TyG-BMI in CKD.

CKD stage	Metabolic heterogeneity	Nutritional status	Dominant signal	Predictive utility	Key confounders	Clinical interpretation
Early (Stages 1–3)	High	Preserved	Metabolic health (TyG)	Strong,U-shaped association with mortality (47)	–	When TyG and BMI are evaluated together, helps differentiate MHO vs. MUO;useful for primary prevention
Advanced (Stages 4–5)	Low (convergent IR)	Depleted (PEW)	Nutritional reserve (BMI)	Weak/Non-significant after adjustment (46)	Fluid overload,inflammation,uremia	Low TyG–BMI signals protein-energy wasting,high mortality risk
Dialysis (ESRD)	Uniformly high	Highly variable,confounded by fluid shifts	Metabolic toxicity (TyG)	Inferior to TyG index alone (51)	Dialysis adequacy,residual renal function,volume status	TyG index preferred;BMI signal unreliable due to volume overload

The evidence from cross-sectional and cohort research is compiled in this table to illustrate how the predictive utility of TyG-BMI evolves across the CKD continuum. In early stages (1–3), high metabolic heterogeneity allows TyG-BMI to differentiate phenotypes (MHO vs. MUO), producing a U-shaped association with mortality (47). Metabolic dysfunction becomes almost universal in advanced stages (4–5) and dialysis, and the dietary signal (low BMI) predominates, resulting in attenuated or inverted correlations (46).In the context of ESRD/dialysis, the use of TyG index alone may be superior to TyG-BMI (51).

The variable performance of TyG-BMI across CKD stages vividly illustrates the dynamic interplay between metabolic health, nutritional reserve, and physical fitness. In early CKD, TyG-BMI captures metabolic heterogeneity; in advanced disease, the convergence toward “low fitness and high metabolic disorder” diminishes its signal. Future risk stratification must therefore integrate staging, body composition, and functional status alongside TyG-BMI.

### 4.4 TyG-BMI,CRMM and CKM

The concept of CRMM is slightly out of date. Marvin and Russell’s 2010 study focused on the relationship between diabetes (metabolic disease), kidney disease, and cardiovascular disease, which is also the forerunner of CRMM (52). Since then, CRMM has progressively gained acceptance and usage.The AHA first proposed the concept of CKM in 2023. The AHA stated that CKM,a systemic disorder that eventually leads to numerous organ dysfunction and a dismal prognosis, is caused by the interaction of CKD, diabetes, obesity, and cardiovascular disease (53). The primary causes of this illness are insulin resistance, persistent inflammation, and aberrant adipose tissue activity. The need for biomarkers that can fully evaluate this multisystem risk is highlighted by the AHA ‘s CKM staging framework (stages 0-4). During the literature period covered in this article (2020–2026), the field has undergone substantial conceptual evolution. Prior to 2023, the academic community typically employed the term “cardio-renal-metabolic multimorbidity (CRMM)” to describe the links among diabetes, cardiovascular disease, and kidney disease. In 2023, the AHA formally introduced the broader concept of CKM, proposing a staging system (Stages 0–4) that highlights the condition’s systemic and progressive nature, as well as its preventability. Accordingly, when assessing earlier work (before 2023), this article retains the original literature’s “CRMM” terminology; when addressing research and stage-based management strategies from 2023 onward, it adopts “CKM” These two terms are not interchangeable but reflect successive phases in the evolving understanding of the disease spectrum.As a result, the TyG-BMI index has emerged as a promising composite indicator that reflects the burden and progression of CRMM and CKM by combining IR with measurements of overall body fat.

#### 4.4.1 CRMM

In depressed CRMM patients, TyG-BMI was involved in disease prediction. A CHARLS study found that patients with greater triglyceride-related indices and depression had the highest incidence of CRMM (54).

A NHANES study revealed that the TyG-BMI showed a positive correlation with cardiovascular and renal disease risk. Despite this, it showed the lowest predictive power of all the TyG composite indicators. In contrast, TyG-WC and TyG-WHtR performed better in identifying cardiovascular and renal disease risks, making them more suitable for early screening and risk stratification. Composite indices involving WC or WHtR should be given priority in future CRMM management (45). Similar findings were observed in another UK Biobank study comparing six IR indicators. The study found that all six IR indicators, including TyG-BMI, were associated with the development and course of CRMM; however, TyG-WC, TyG-WHtR, and the Metabolic Score for Insulin Resistance (METS-IR) performed better (55). Another UK Biobank-based study that examined the relationship between the single marker and CRMM found that TyG-BMI significantly influenced the onset and course of CRMM (56). While TyG-BMI demonstrates predictive value, emerging indicators like TyG-WC and TyG-WHtR currently show superior predictive performance in certain aspects.

Similar to the DKD study, some research has reached opposing conclusions. According to a prospective cohort study that looked at 5,845 adult Iranians in the Tehran Lipid and Glucose research (TLGS) cohort, TyG-BMI did not provide a better advantage over the TyG index (57).

In conclusion, the fundamental reason for TyG-BMI’s insufficient predictive performance in CRMM lies in the inherent strength of the TyG index, which accurately depicts IR, the primary pathophysiological mechanism of CRMM. Meanwhile,BMI ‘s ability to measure the distribution of body fat is limited. While BMI can only approximate the distribution of body fat, WC and WHtR, two indicators combined with waist circumference, can more accurately reflect the features of “central obesity” in patients and show better predictive value. The development and occurrence of CRMM are more strongly associated with visceral fat gain. In order to avoid the drawbacks of a single indicator, a combination of several indicators should be used in clinical applications. In an attempt to perform a thorough and methodical analysis of the patient’s status, it may even be necessary to create a comprehensive scoring system.According to our earlier analysis, BMI’s predictive ability declines because it is less accurate than WC and WHtR in capturing “body composition and fitness measures” of body health status such muscle mass, visceral fat thickness, and cardiorespiratory endurance (**Figure 1**).

**Figure 1:**
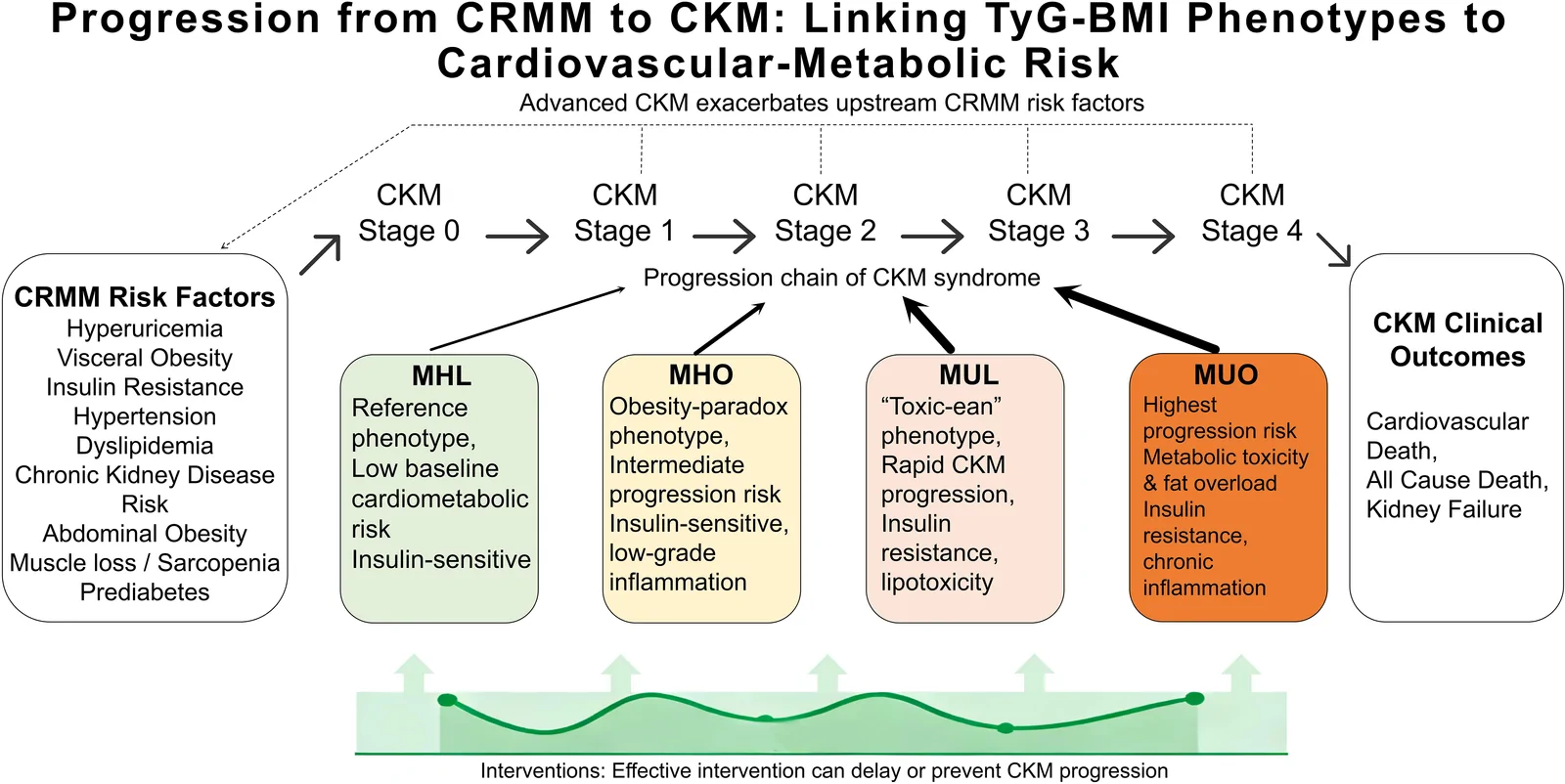
Progression from CRMM to CKM syndrome. CRMM represents the coexistence of CKD, cardiovascular disease and metabolic disease (diabetes/obesity). The AHA’s 2023 CKM staging framework (phase 0-4) formally established this interaction, emphasizing the core role of pathological states such as insulin resistance, chronic inflammation and adipose tissue dysfunction. Our metabolic phenotype reflects this interaction to some extent, and early intervention can delay this progress to some extent.

#### 4.4.2 CKM syndrome

Under various circumstances, TyG-BMI shows potential in predicting the prognosis of CKM patients.Elevated TyG-BMI corresponds with CKM, according to the CHARLS study on CKM risk in patients with chronic liver disease (CLD) (58). An essential organ for controlling the body’s metabolism is the liver.This suggests that monitoring TyG-BMI could enhance CKM prognosis in CLD patients,However, it should be mentioned that the consequences of various forms of CLD may differ.Since we observed the effect of NAFLD on TyG-BMI in the study of the relationship between TyG-BMI and DKD, more comprehensive research is needed to clarify the relationship between TyG-BMI and specific types of liver disease patients (35).

The prognostic monitoring of TyG-BMI is also affected by the various stages of CKM syndrome. An L-shaped association between TyG-BMI and all-cause mortality was found in an NHANES study examining the relationship between triglyceride-glucose-related indices and cardiovascular and all-cause mortality in CKD patients with CKM syndrome stages 0–3. Stages 1 and 3 showed stronger relationships. However, it was discovered that TyG-WHtR and TyG-WC were more accurate predictors; this is consistent with some studies before TyG-BMI (59). When the study expanded to include CKM syndrome patients from stages 0-4, another NHANES study revealed that TyG-BMI had independent predictive value for diabetic mortality in CKM syndrome patients, but showed lower predictive power for death from cardiovascular disease and all causes. All-cause mortality exhibited a U-shaped relationship (nonlinear) with TyG-BMI, with the lowest risk point at TyG-BMI/10 = 20.21. Cardiovascular disease mortality also followed a U-shaped relationship (nonlinear), with the lowest risk point at TyG-BMI/10 = 18.61. There were no notable inflection points in the linear positive connection between diabetes mortality and TyG-BMI. TyG-BMI may be helpful as a therapeutic reference in some populations, as evidenced by its inconsistent relationships with age, gender, and metabolic status (60). Compared to patients in stages 0-3, the observed association changes may be attributed to the inclusion of more advanced and complex cases. A MIMIC-IV database study fills this gap by revealing that in patients diagnosed with stage 4 CKM syndrome, reduced TyG-BMI index significantly correlates with increased all-cause mortality risks within 180 days and one year. As a result, the TyG-BMI score is a helpful tool for risk stratification and prognosis assessment in this patient population (61). The “obesity paradox”—a low BMI in patients with serious illnesses suggests lower nutritional status—is further revealed by this study. As all participants were stage 4 CKM syndrome patients under intensive care, their lower BMI reflected advanced clinical deterioration. Surprisingly, the research demonstrated that reduced TyG-BMI correlates with increased mortality. This explains why the predictive power of TyG-BMI diminished when the study scope expanded from stages 0–3 CKM syndrome to stages 0–4 patients, with the correlation strength also decreasing.

In patients with CKM syndrome, TyG-BMI also predicts the incidence of cardiovascular disease.The findings from several investigations carried out at various locations are very similar. Two studies utilizing the CHARLS database all demonstrated a positive correlation between TyG-BMI and the incidence of cardiovascular disease in patients with cardiorenal metabolic syndrome. Patients with CKM syndrome in stages 0–3 might use the TyG-BMI index as a monitoring tool (62, 63). A follow-up GOLD-Health cohort study further investigated eight IR indices, including TyG-BMI. All eight indices demonstrated predictive significance for cardiovascular disease in CKM syndrome patients; TyG-WC had the highest predictive ability (64). In a comparable NHANES study assessing 16 baseline overweight and lipid-related indices for cardiovascular disease to predict risk in individuals with CKM syndrome stages 0–3, TyG-BMI showed predictive efficacy, but the novel index TyG-WC outperformed it (65). TyG-BMI ‘s primary drawback is that, unlike WC, BMI is not a reliable indicator of fat distribution.

A study from the UK Biobank compared TyG-related indicators, revealing comprehensive insights into disease risk and adverse prognosis, and established thresholds: In individuals with stages 0–3 of CKM syndrome, TyG-BMI may serve as an effective indicator of cardiovascular disease mortality and incidence risk.It shows a positive correlation with cardiovascular disease risk, with a sharp increase after surpassing specific inflection points. Incorporating TyG-BMI into traditional risk factors significantly enhances risk stratification capabilities, aiding in early identification and intervention of high-risk individuals. The study revealed nonlinear relationships (U-shaped or J-shaped) between TyG-BMI and multiple outcomes, with clear risk inflection points: All-cardiovascular disease: inflection point at TyG-BMI = 322.135; Coronary Heart Disease (CHD): inflection point at TyG-BMI = 279.954; All-Cause Mortality: inflection point at TyG-BMI = 196.234; Cardiovascular Mortality: inflection point at TyG-BMI = 179.138 (66). Additionally, another NHANES study found that TyG-ABSI showed a higher connection with mortality risk in those with CKM syndrome stages 0–3 (67). These watershed moments also serve as a reminder that the “obesity paradox” exists objectively. We should make every effort to thoroughly assess several signs when using them for prediction.Additionally, we anticipate more extensive research to identify more thorough indicators and elucidate their application scenarios.

CKM has a staging system, just as CKD, and patients in different stages have varied metabolic states. Based on the phenotypic framework established in Section 3, future research could explore stage-adapted management strategies, as outlined in **Table 4**, which presents hypotheses requiring validation in prospective cohorts and randomized trials.

**Table 4:** Stage-specific hypothetical framework for TyG-BMI in CKM syndrome: directions for future research.

Different CKM stages	Potential research focus/hypothesis
High TyG-BMI	Hypothesis: Patients with high TyG-BMI (metabolically unhealthy obesity) may benefit from targeted metabolic health improvement. Future studies should test whether interventions such as SGLT2 inhibitors, GLP-1 receptor agonists, and lifestyle modifications—with established cardiorenal benefits in broader populations—can improve outcomes specifically in this TyG-BMI-defined subgroup.
Moderate TyG-BMI	Hypothesis: Patients with a moderate TyG-BMI (a potential MHO phenotype) may gain greater long-term survival benefits by maintaining metabolic stability rather than pursuing aggressive weight loss. Prospective studies are needed to assess whether exercise training designed to improve muscular and cardiorespiratory fitness can help sustain the transient “paradoxical” advantage and prevent metabolic deterioration.
Low TyG-BMI	Hypothesis: Low TyG-BMI in advanced disease may signal protein-energy wasting and an extremely high risk of short-term mortality. Interventional studies should explore whether targeted nutritional support can mitigate this risk, while acknowledging that the primary driver of mortality may be the underlying catabolic state rather than modifiable nutrition.

Based on the phenotypic framework established in Section 3, this table proposes stage-adapted management strategies informed by evidence from NHANES (59, 60, 65, 67), CHARLS (62, 63), and MIMIC-IV (61) studies. In early stages (0–3), elevated TyG-BMI, when accompanied by both high TyG and high BMI, identifies MUO phenotype warranting metabolic intervention (e.g., SGLT2 inhibitors, lifestyle modification). In stage 4, low TyG-BMI signals nutritional depletion and high short-term mortality risk, necessitating supportive care. The intermediate TyG-BMI range may represent MHO phenotype, where preserving fitness and preventing metabolic deterioration are priorities.

A particularly intricate pathophysiological scenario for comprehending the TyG-BMI and obesity paradox is provided by CKM. TyG-BMI effectively captures the dynamic balance between “metabolic toxicity” and “nutritional reserve” as a composite indicator. According to the theory presented in the Nature review, “fitness” is the most important factor in controlling this equilibrium. To attain genuine precision medicine, future CKM syndrome research and management must incorporate thorough evaluations of body composition, physical fitness, and metabolic health.

### 4.5 TyG-BMI and RRT

A national survey indicates that China’s dialysis patient prevalence rate rose from 255.From 11 in 2013 to 419.39 in 2017.It is anticipated that the average number of events per million people (pmp) will continue to rise (68). As dialysis patient numbers increase, monitoring dialysis-related complications has become a critical priority, with TyG-BMI showing significant research potential.

#### 4.5.1 Peritoneal dialysis

Peritoneal dialysis (PD), a crucial at-home treatment for renal failure, accounts for 11% of dialysis cases globally and 9% of all renal replacement therapies (69). TyG-BMI also shows potential as a monitoring tool for PD patients.

A study involving 865 patients using RCS analysis revealed a U-shaped association between TyG-BMI and all-cause mortality in PD patients, while showing a J-shaped relationship with cardiovascular mortality. The all-cause mortality inflection point for TyG-BMI was 209.73, while the cardiovascular disease mortality inflection point was 206.64 (70). Another similar study with 2,335 participants indicated that elevated TyG-BMI was independently associated with greater risk of cardiovascular disease and all-cause death in PD patients (71).

1,736 people were included in a study that looked at the connection between IR markers and all-cause mortality in PD patients. TyG index, TyG-BMI, and triglyceride/high-density lipoprotein cholesterol (TG/HDL-C) were among the non-insulin-based IR surrogate measures that were assessed in the study. The TyG index turned out to be the most reliable substitute for IR, and the results showed a significant correlation between TyG-BMI and all-cause mortality in individuals with PD (51). The finding that the TyG index alone outperforms TyG-BMI for mortality prediction in PD patients provides a striking validation of the primacy of metabolic health over mere weight status in advanced illness. In the cachectic milieu of ESRD, BMI becomes an unreliable indicator of nutritional health due to fluid shifts and wasting. The TyG index, as a pure marker of IR, emerges as the dominant prognostic factor. This result is consistent with Section 3.4’s conceptual framework: when “fitness” (here, metabolic fitness proxied by TyG) is very low, the obesity paradox emerges, but fitness itself remains the dominant risk modifier.

#### 4.5.2 Hemodialysis

Hemodialysis (HD), the most popular kind of RRT worldwide, accounts for about 69% of kidney replacement therapies and 89% of dialysis cases (72). TyG-BMI in HD patients has been studied from a variety of angles. According to a study that looked at 219 HD patients, TyG-BMI is an independent risk factor for coronary artery calcification (CACS) and there is a positive correlation between TyG-BMI and CACS in maintenance HD (MHD) patients (73). Another study expanded its scope to investigate the relationship between emerging IR indicators—such as TyG-BMI, fasting blood glucose to HDL-C ratio, and platelet to HDL-C ratio—with abdominal aortic calcification(AAC) in HD patients. TyG-BMI was found to be independently associated with AAC in MHD patients (74).

TyG-BMI has also been preliminarily studied in relation to the maturity of arteriovenous fistulas (AVFs). A study involving 249 patients found that TyG-BMI is an independent predictor of AVF maturity, providing a new reference measure for monitoring AVF development (75).

#### 4.5.3 Kidney transplantation

The significance of TyG-BMI in KT has also been investigated as the procedure becomes more widespread.In kidney transplant recipients, TyG-BMI is a unique predictor of post-transplant diabetes mellitus (PTDM).Among several metabolism-related markers, TyG-BMI was determined to be the best replacement marker for predicting PTDM, highlighting the importance of assessing both IR and weight status following transplantation (76).

### 4.6 Methodological limitations of current evidence: a critical appraisal

A substantial proportion of high-quality evidence on TyG-BMI and kidney disease derives from secondary analyses of public databases (e.g., NHANES, CHARLS, UK Biobank). While valuable, this evidence base is constrained by several methodological limitations that contribute to the heterogeneous findings reviewed above.

Population heterogeneity: Geographic and ethnic differences—for instance, NHANES (U.S. population) versus CHARLS (middle-aged/older Chinese adults)—lead to inconsistent association strengths. The superior predictive performance of TyG-BMI in CHARLS (62, 63) compared to NHANES (59, 60, 65, 67) may reflect true biological differences in fat distribution and metabolic sensitivity rather than indicator superiority.

Measurement error: Biological variability in fasting glucose and triglycerides, coupled with volume overload in CKD patients distorting BMI, introduces regression dilution bias. This may explain why indices incorporating central obesity (TyG-WC, TyG-WHtR) consistently outperform TyG-BMI, as waist circumference is less susceptible to fluid shifts.

Confounding and over-adjustment: Unmeasured confounders (diet, physical activity) and inappropriate adjustment for mediators (hypertension, proteinuria) can obscure true associations. The loss of significance for TyG-BMI after multivariable adjustment (46) may reflect over-adjustment, but could also stem from insufficient sample size, true absence of independent predictive value, or confounding by disease severity. Without a formal causal framework (e.g., directed acyclic graphs), it is difficult to adjudicate among these possibilities. Variables such as hypertension and proteinuria may act as confounders, mediators, or markers of disease severity depending on the specific research question.

Reverse causality: Kidney disease itself induces metabolic dysfunction (e.g., uremia exacerbating IR), creating bidirectional relationships that cross-sectional analyses cannot resolve.

In addition, the four-quadrant metabolic phenotype framework proposed in this study has a key methodological limitation in its application: the inherent indecomposability of TyG-BMI as a single composite index. As noted earlier, because this index is the product of TyG and BMI, its value alone cannot uniquely determine an individual’s metabolic health status or nutritional reserve status. This implies that in existing studies, reporting only the overall TyG-BMI level without disclosing its component composition may lead to oversimplification or even misinterpretation of the “obesity paradox.” Future research should prioritize the use of multivariate models that include TyG and BMI as independent covariates, or explicitly report stratified results based on the respective cutoffs of the two indices, in order to more accurately capture the heterogeneity of metabolic phenotypes.

These limitations lead to conflicting results and hinder clinical translation. Future research should prioritize multicenter prospective cohort studies, standardized protocols, direct body composition assessments, and the differentiation of specific phenotypes to validate and extend existing findings.

Despite these limitations, the accumulated evidence points to specific contexts in which TyG-BMI holds particular advantages. First, when waist circumference is unavailable—a frequent constraint in large-scale epidemiological datasets (e.g., CHARLS, UK Biobank baseline)—TyG-BMI is the only composite IR index that integrates both metabolic and nutritional dimensions. Second, in early-stage CKD and general population screening, where metabolic heterogeneity is greatest, the dual-signal nature of TyG-BMI (TyG for metabolic toxicity, BMI for nutritional reserve) yields more comprehensive risk capture than TyG alone. Third, in specific T2DM subgroups, such as patients without NAFLD or those classified as having severe insulin-resistant diabetes (SIRD), TyG-BMI outperforms other indices in predicting DKD. Conversely, in advanced CKD, dialysis, ICU settings, and CKM stage 4, where BMI is confounded by fluid overload, muscle wasting, or catabolic dominance, TyG-BMI should be replaced by the TyG index, TyG-WC, or TyG-WHtR, which better isolate metabolic dysfunction.

## 5 Conclusion and future perspectives

TyG-BMI is a pragmatic, composite marker that captures both dysmetabolism (TyG) and nutritional status (BMI). It holds promise as a screening and phenotyping tool in metabolically heterogeneous populations (e.g., general population, early CKD, specific T2DM subgroups). Its unique ability to reflect the ‘obesity paradox’ through the integration of its two components (TyG and BMI) provides valuable insights into the altered risk dynamics in advanced illness.

However, its predictive utility is not universal and is modulated by several factors. Major limitations include: ① Frequent non-linear (U-/J-/L-shaped) associations. ② Variable performance across patient subgroups and disease stages. ③ Systematic inferiority to indices of central obesity (TyG-WC, TyG-WHtR) for disease incidence prediction. ④ Attenuation in advanced disease where metabolic dysfunction becomes universal.

Ultimately, 46 original studies meeting the eligibility criteria were included, covering five major fields: AKI,DKD,CKD,CRMM/CKM and RRT. To facilitate visual comparison, we constructed an evidence table (**Supplementary Table 1**) that systematically collates the design type, cohort source, sample size, target population, kidney-related outcomes, TyG-BMI modeling approach (continuous, categorical, or restricted cubic spline [RCS]), key adjusted variables, fully adjusted effect estimates, non-linear dose–response findings, comparisons with alternative insulin resistance indicators, and major methodological limitations of each study. This table allows readers to rapidly survey the existing evidence, identify similarities and differences in the TyG-BMI–renal outcome association across disease stages and populations, and pave the way for subsequent stratified analyses.

In the future, TyG-BMI still has to be improved in a number of areas:

### 5.1 Moving beyond the “one-size-fits-all” threshold

Different thresholds/cut-off values were mentioned in the studies we collected. We briefly analyzed the different thresholds and formed **Table 5**.

**Table 5:** Cross-study standardized analysis of TyG-BMI and related index thresholds.

Document number	Formula/scale	Study population (country, n)	Endpoints	Modeling method	Threshold derivation method	Threshold/cutoff value	Performance evaluation metrics	Comparison indicators (core conclusions)
(25)	TyG-BMI	US SA-AKI ICU (3,349)	28-day all-cause mortality	Cox + RCS	RCS L-shaped Inflection Point	261.7	HR 0.998/unit; T3 vs T1 HR 0.68	CRRT subgroup interaction p=0.001; low value paradoxical high risk
(32)	TyG, TyG-BMI, HOMA-IR, METS-IR	US DKD (1,588)	All-cause mortality	Cox + RCS + piecewise regression	RCS + Segmented Regression U-shaped Inflection Point	TyG 9.18; TyG-BMI 207.9	AUC 0.789–0.791; well calibrated	Four indicators AUC comparable; only TyG Q4 significantly increases mortality risk
(36)	TyG, TyG-BMI	Chinese Newly Diagnosed T2DM (924)	DKD prevalence	Logistic + RCS + ROC	RCS Inflection Point + Youden Cutoff	RCS: TyG 9.68, TyG-BMI 243; ROC: TyG 10.08, TyG-BMI 221.5	AUC 0.571 (TyG), 0.553 (TyG-BMI)	TyG superior to TyG-BMI; both have poor diagnostic value
(46)	TyG, TyG-BMI, TyG-WHtR, METS-IR, HOMA-IR	US General Population (10,660)	CKD prevalence	Logistic + RCS + ROC	RCS U-shaped Inflection Point	TyG 9.05; TyG-BMI 245; TyG-WHtR 4.3	AUC 0.630 (TyG-WHtR highest)	TyG-WHtR optimal (highest AUC + only significant predictor of mortality)
(47)	TyG-BMI, TyG	US CKD (3,089)	All-cause/CVD mortality	Cox + RCS + ROC	RCS Inflection Point + Youden Cutoff	RCS: all-cause 299.31, CVD 294.85; ROC: all-cause 272.57, CVD 280.34	AUC 0.574 (TyG-BMI) vs 0.527 (TyG)	TyG-BMI superior to TyG; more significant in elderly subgroup
(48)	TyG, TyG-BMI, TG/HDL-c, METS-IR	Taiwan CKD Stage 1-4 (2,457)	Renal outcomes + all-cause mortality	Cox quartile	Quartiles (No RCS/ROC)	No single inflection point; low TyG-BMI group HR 1.87 (all-cause)	AUC not reported	Low TyG/TyG-BMI paradoxical high risk; only high TyG predicts renal outcomes
(49)	TyG, TyG-BMI, TyG-WC, TyG-WHtR	China CHARLS Middle-aged and Elderly (3,037)	MCD incidence	Cox + RCS + ROC	RCS Inflection Point + Youden Cutoff	RCS: TyG-BMI 171, TyG-WC 609, TyG-WHtR 3.83; ROC: model probability cutoff 0.248, etc.	AUC 0.610 (TyG) ≈ 0.607 (TyG-WHtR)	TyG and TyG-WHtR have comparable predictive value; TyG linear, combined indicators nonlinear
(59)	TyG, TyG-BMI, TyG-WC, TyG-WHtR	US CKM Stage 0-3 (6,383)	All-cause/CVD mortality	Cox + additive model + two-stage Cox	Two-segment Cox L-shaped Inflection Point	TyG-WHtR: all-cause 3.74, CVD 6.38; TyG-BMI: all-cause 169.05	Per SD HR: TyG-WC 1.50, TyG-WHtR 1.45	TyG-WC optimal (all-cause), TyG-WHtR optimal (CVD); strongest effect in CKM stage 1/3
(66)	TyG, TyG-BMI, TyG-WC, TyG-WHtR	UK Biobank CKM Stage 0-3 (282,920)	CVD incidence + mortality	Cox + RCS + two-stage Cox + NRI/IDI	RCS + Two-segment Cox J-shaped Inflection Point	TyG: all-cause 8.691; TyG-BMI: CVD 322.135, all-cause 196.234; TyG-WHtR: CVD 5.267, all-cause 4.695	AUC Δ=0.0017–0.0025; IDI 0.160 (TyG-WHtR)	TyG combined indicators superior to single index; TyG-WHtR highest IDI, TyG-WC highest NRI
(70)	TyG-BMI	China PD (865)	All-cause/CVD mortality	Cox + RCS + tertile	RCS U/J-shaped Inflection Point	all-cause 209.73; CVD 206.64	High vs medium HR 1.84 (all-cause), 2.94 (CVD)	Gender interaction p=0.016–0.046; middle group optimal

The critical TyG-BMI values reported in the ten studies cited above cover a broad range (169–322), and direct comparison may yield misleading inferences. Systematic analysis reveals that the heterogeneity stems mainly from three dimensions:

#### 5.1.1 Ethnic and metabolic baseline differences

The TyG-BMI inflection points in Asian populations (PD 209.73, DKD 207.9, CKD 245 in China) are significantly lower than those in Western populations (CKD 299.31 in the United States, Cardiovascular Disease[CVD] 322.135 in the United Kingdom). This difference is largely driven by baseline BMI disparities: average BMI in Asian cohorts is approximately 21–25 kg/m², while in Western cohorts it is about 28–32 kg/m². Because TyG-BMI is the product of TyG and BMI, the multiplicative contribution of BMI directly elevates the absolute values.

#### 5.1.2 Endpoint definition and disease severity

Chronic disease risk (CKD, MCD) displays low inflection points (171–245), reflecting the cumulative effects of long-term metabolic disturbances. Severe short-term death (SA-AKI 28-day mortality) exhibits an L-shaped inflection at 261.7, with paradoxically elevated risk at low values, indicating an amplified “obesity paradox” in catabolic states. Cardiovascular events and deaths (CVD onset, CVD death) have the highest inflection points (280–322), because atherosclerosis requires a greater insulin resistance burden to be triggered. End-stage renal disease (PD) all-cause mortality follows a U-shaped curve with an inflection at 209.73, where the middle group has the most favorable prognosis, reflecting the bidirectional risk of malnutrition–inflammation–cachexia syndrome.

#### 5.1.3 Fundamental differences between statistical modeling methods and threshold derivation paradigms

The RCS inflection point [references (6, 25, 32, 36, 47, 49, 59, 66, 70)] identifies the “risk transition point” of the association, reflecting the boundary between increasing and decreasing risk in nonlinear relationships.

The ROC/Youden cutoff [references (36, 47, 49)] seeks the “optimal diagnostic cut-off point” that maximizes the sum of sensitivity and specificity.

The two Cox critical values [references (59, 66)] employ the likelihood ratio test to locate a “risk ratio breakpoint,” constituting a third paradigm.

In the same study [e.g., reference (47)], the RCS inflection point (299.31) and the ROC cutoff (272.57) differ by 26.74 units, directly demonstrating the numerical incomparability introduced by different methods.

Therefore, cross-study comparisons require clarifying threshold semantics first and then conducting a hierarchical analysis by method.

Standardization suggestion: When reporting TyG-BMI thresholds in the future, ① clearly label the derivation method and semantics; ② prioritize dimensionless indicators (TyG single index or TyG-WHtR) for cross-population comparisons; and ③ conduct a meta-regression based on individual participant data (IPD), with baseline BMI, ethnicity, and endpoint type as covariates.

### 5.2 Expanding research scope

As an emerging IR indicator, the role of TyG-BMI in other kidney diseases remains unclear. For instance, only one study has looked at the connection between left ventricular hypertrophy and IgA nephropathy (IgAN), the most prevalent glomerular disease in the world (77), However, there is still a dearth of studies on kidney injury in patients with IgAN.IgAN has significant ethnic differences, as is well known. Compared to populations in Europe and the US, Asian populations have a much higher prevalence of IgAN, and early detection and treatment are crucial in lowering the burden. Similarly, studies on common conditions like glomerulonephritis and nephrotic syndrome are also scarce. To fully understand the connection between TyG-BMI and these illnesses, more research is desperately needed.Metabolic abnormalities, particularly dyslipidemia, are frequently associated with nephrotic syndrome, and the role of TyG-BMI in nephrotic syndrome has not yet been investigated.

### 5.3 Leveraging strengths in multisystem disease

As a biomarker that simultaneously measures lipid-glycemic metabolic disorders and nutritional status, TyG-BMI shows a strong correlation with metabolic-associated kidney disease (MAKD), a multidisciplinary focus in current research. A study found that in people with type 2 diabetes, TyG-BMI is associated with biomarkers related to the heart, kidneys, liver, and bones (78). It is expected that more investigation will shed light on the connection between multi-system consequences of kidney illness and TyG-BMI.Today’s illnesses are more often systemic than single-system illnesses. The multi-angle indicators of the body found in the TyG-BMI itself are useful for analyzing diseases affecting multiple systems.

### 5.4 Developing next-generation metrics

Develop indicators that better integrate visceral fat parameters or direct body composition analysis, to comprehensively upgrade TyG-BMI.In order to support accurate kidney disease diagnosis and treatment, a more thorough index system may be created in the future to create a comprehensive scoring system to evaluate patients’ disease status and finally achieve precise risk classification.

### 5.5 Integrating fitness and body composition

The ‘obesity paradox’ framework established in this review highlights that prognosis depends on the dynamic balance among metabolic health, nutritional reserve, and physical fitness. Future research must strive to incorporate direct measures of muscle mass, cardiorespiratory fitness, and functional status alongside TyG-BMI. Only through such multidimensional assessment can we achieve personalized risk stratification in kidney disease.

### 5.6 Scenario-indicator recommendation

Under different conditions, different indicators have different scope of application. We compared these new indicators and briefly analyzed the scope of application of different indicators, but the precise scope of application still needs to be determined by large-scale research (**Table 6**).

**Table 6:** Application scenarios for TyG-BMI and other indicators.

Clinical/research scenario	Recommended indicators	Reason
When waist circumference data is unavailable (e.g., large retrospective databases only have height, weight, fasting blood glucose, triglycerides)	TyG-BMI	Only composite IR indicator that is calculable; BMI still holds value as a nutritional reserve signal
General population or early CKD screening (eGFR ≥ 60 ml/min/1.73m²)	TyG-BMI or TyG-WC	BMI is easily obtainable, and metabolic disturbances are not yet extreme at this stage; TyG-BMI can effectively capture risk; if WC is available, TyG-WC is superior
Specific T2DM subgroups (e.g., patients without NAFLD, SIRD subtype)	TyG-BMI	In this subgroup, TyG-BMI independently predicts DKD risk and outperforms other indicators (25, 29)
Advanced CKD (G4-G5), dialysis patients (PD/HD)	TyG index or TyG-WHtR	BMI is heavily confounded by volume load and muscle wasting; TyG index, as a pure metabolic marker, performs best (42)
ICU/AKI/critically ill patients (e.g., SA-AKI, CKM stage 4)	TyG-WC or TyG-WHtR	Central obesity indicators better reflect visceral fat and metabolic toxicity, and are not affected by fluid retention (14, 15, 51)
CKM syndrome staging management (stage 0–3 vs stage 4)	Stage 0-3: TyG-BMI combined with TyG-WC Stage 4: TyG index or TyG-ABSI	Metabolic heterogeneity still exists in stages 0-3, requiring dual indicator complementarity; stage 4 is dominated by catabolism, necessitating pure metabolic indicators (49–51, 57)

The TyG-BMI index may be more useful when waist circumference cannot be measured, during early CKD screening, or in certain T2DM subgroups such as patients with non-alcoholic fatty liver disease or SIRD. Conversely, in patients with advanced CKD requiring dialysis, those who are critically ill, or individuals with CKM stage 4, alternative indices such as the TyG index, TyG-WC, or TyG-WHtR should be prioritized, as fluid overload and catabolic states introduce.

In conclusion, the integration of insights from Lavie et al.’s seminal work (7) allows for a profound reinterpretation of TyG-BMI’s role in kidney diseases. It transcends being a simple risk calculator and becomes a tool for phenotyping patients based on their metabolic health and nutritional status when TyG and BMI are considered jointly. The frequent non-linear relationships observed across the spectrum of kidney diseases are not mere statistical anomalies but may reflect the dynamic tension between the detrimental effects of IR and the protective effects of metabolic and nutritional reserves, a concept at the very heart of the ‘obesity paradox’. Future research must strive to integrate direct measures of body composition (to differentiate fat from muscle mass) and, ideally, cardiorespiratory fitness​into this framework. By doing so, we can move beyond the limitations of BMI and TyG-BMI alone, towards a truly personalized assessment that captures the complex interplay of fatness, fitness, and metabolic health in determining the prognosis of patients with kidney disease.
