# Supplementary material for: Insulin resistance, metabolic phenotypes, and the obesity paradox: a triglyceride glucose-body mass index framework in partial kidney diseases
Source: Front Endocrinol (Lausanne). 2026 Sep 23;17:1915011. doi: 10.3389/fendo.2026.1915011 (PMC13643709; doi:10.3389/fendo.2026.1915011)
Supplement: Supplementary file 1 [file Table1.docx]

Supplementary Table 1

Research Evidence Table

| NO . | Disease category | research design | Queue/database | sample size | Target population | Kidney/primary outcome | TyG-BMI modeling | Key adjustment factors | Main effect estimation (highest vs. lowest group) | Nonlinear/threshold discovery | Key comparison indicators | Main limitations |
| --- | --- | --- | --- | --- | --- | --- | --- | --- | --- | --- | --- | --- |
| 6 | CKD | Cross sectional study | NHANES | 10,660 | Adults over 20 | CKD prevalence and mortality | Continuous variable, quartile, RCS | Age, gender, race, BMI, smoking, hypertension, hyperuricemia, etc | OR=0.92 (Q4 vs Q1, P-trend=0.048) | U-shaped nonlinearity (P<0.0001) Inflection point: 245 | Predictive power is significantly weaker than TyG-WHtR | Cross sectional design, American population only |
| 22 | AKI | Multicenter retrospective cohort | MIMIC-IV, eICU-CRD | 3,550 | ICU patients with heart failure | AKI risk | Continuous variable, quartile, RCS | Age, gender, triglyceride, hemoglobin, NT proBNP, sepsis, etc | HR=1.84 (Q4 vs Q1) | Linear positive correlation (P-nonlinear=0.091) | Better than TyG or BMI alone | Retrospective bias, single baseline measurement, ICU only |
| 24 | AKI | Multicenter retrospective cohort | MIMIC-IV | 1,117 | Septic ICU patients | AKI, RRT risk | Continuous variable (per 10 units), quartile, RCS | Age, gender, SOFA, SIRS, infection site, multiple laboratory indicators | AKI: HR=1.49  RRT: HR=2.50 (Q4 vs Q1) | Nonlinear (AKI&RRT P-nonlinear<0.05) | Improve the prediction efficiency of traditional score (SOFA, SAPS II) | Retrospective bias, lack of inflammatory markers (PCT, CRP) |
| 25 | AKI | Retrospective cohort | MIMIC-IV | 3,349 | Patients with sepsis related AKI | Short term mortality (28 days, 90 days, 180 days) | Continuous variable, quartile, RCS | Age, race, Charlson index, SOFA, SAPS II, vital signs, laboratory indicators | 28 day mortality: HR=0.69 (T3 vs T1) | L-shaped nonlinearity (P<0.001) Inflection point: 261.7 | The protective effect comes from BMI components ("obesity paradox") | Retrospective bias, large amount of data missing |
| 27 | AKI | Single center retrospective cohort | Electronic medical record of Shanghai Oriental Hospital | 126 | Diabetic patients after CABG | AKI risk | Continuous variable, logistic regression | Preoperative albumin, CKD history, preoperative creatinine, ICU stay, etc | OR=1.03 (per unit increment) | Linear (without RCS) | Improve the traditional clinical model (AUC 0.766 → 0.836) | Single center, small sample, single measurement |
| 28 | AKI | Single center retrospective cohort | Electronic medical record of Hebei Provincial People's Hospital | 1,041 | Non diabetic STEMI patients undergoing emergency PCI | Contrast induced nephropathy (CIN) risk | Continuous variable, logistic regression | Total cholesterol, LDL-C, creatinine and uric acid on the 3rd day after operation | OR=1.029 (per unit increment) | Linear (without RCS) | Better than TyG and METS-IR (AUC 0.812) | Single center, single measurement, lack of dynamic observation |
| 29 | AKI | Single center retrospective cohort | Electronic medical records of Inonu University | 980 | Non diabetic STEMI patients undergoing emergency PCI | CIN risk | Continuous variable, logistic regression | CRP, Baseline creatinine, uric acid, contrast agent dosage | OR=1.014 (per unit increment) | Linear (without RCS) | Better than TyG (AUC 0.676 vs 0.600) | Single center, small sample number of events (120 cases) |
| 32 | DKD | Retrospective cohort | NHANES | 1,588 | DKD patients | All cause mortality | Continuous variable, quartile, RCS | Age, gender, race, BMI, liver and kidney function, blood lipids, cardiovascular disease | HR=1.01 (continuous variable, P=0.005) | U-shaped nonlinearity (P<0.001) Inflection point: 207.9 | It has the same predictive power as other IR indicators (HOMA-IR, TyG) | Cross section data, unable to reflect dynamic changes |
| 33 | DKD | Cross sectional study | Zhongda Hospital Affiliated to Southeast University | 1,080 | T2DM patients | Albuminuria/CKD risk | Continuous variable, logistic regression | Gender, age, history of hypertension, HDL-C, LDL-C, HbA1c | Albuminuria: OR=1.004 CKD: OR=1.005 (per unit increment) | Linear (without RCS) | No formal comparison of other indicators | Cross section, cause and effect cannot be established |
| 34 | DKD | Cross sectional study | NHANES | 6,891 | Diabetics | Risk of diabetic nephropathy (DN) | Continuous variable, quartile, RCS | Gender, age, race, education, smoking, hypertension, coronary heart disease, etc | OR=1.57 (Q4 vs Q1) | Nonlinear effect (P value is less than 0.001 on the whole, and P value of nonlinear effect is 0.039) Inflection point: 288.55 | Less predictive than TyG index | Cross section design, cause and effect cannot be inferred |
| 35 | DKD | Cross sectional study | The First Affiliated Hospital of University of science and technology of China | 425 | T2DM patients (stratified by NAFLD) | DKD risk | Continuous variable, logistic regression | Age, gender, duration of diabetes | OR=1.025 (per unit increment) | Linear (without RCS) | In the subgroup without NAFLD, it was better than TyG (AUC 0.854) | Small sample, cross section, lack of drug information |
| 36 | DKD | Cross sectional study | The Second Affiliated Hospital of Nanchang University | 924 | Newly diagnosed T2DM (without medication) | DKD risk | Continuous variable, quartile, RCS | Age, gender, family history, smoking, drinking, HbA1c, blood lipids, uric acid | OR=1.83 (Q4 vs Q1, coarse model) | Linear (P-nonlinear=0.055) | Predictive power was weaker than TyG (AUC 0.553 vs 0.571) | Single center, cross-sectional, inpatient bias |
| 37 | DKD | Retrospective cohort study | Third Affiliated Hospital of Soochow University | 521 | Newly diagnosed T2DM adults | DKD risk | Continuous, tertiles, ROC, C-statistics, NRI, IDI (TyG, TyG-BMI, TG/HDL, METS-IR) | Age, sex, smoking, hypertension, SBP, FCP, UACR, eGFR, UA, TC, ApoB, FT4, DR, DPN, antihypertensive treatment | TyG OR = 4.936; TyG-BMI OR = 3.467; TG/HDL OR = 4.693; METS-IR OR = 4.310 (all P < 0.001) | Positive linear association(RCS/nonlinearity not reported in main text) | TyG & TG/HDL C-stat = 0.652; all alternative indexes improved baseline prediction (positive NRI/IDI) | Single-center, small sample, Chinese only |
| 38 | DKD | Cross sectional study | Shandong Provincial Hospital | 1,026 | T2DM patients | DKD risk | Continuous variable, quartile | Gender, age, smoking, drinking, course of disease, BMI, waist circumference, blood pressure, uric acid, HbA1c | OR=0.84 (Q4 vs Q1, P=0.658) | Linear (without RCS) | The predictive power was the worst (AUC 0.599), much lower than TyG (0.755) | Cross section, Chinese inpatients only |
| 39 | DKD | Prospective cohort | Copernican queue Copernicus queue | 567 | Newly diagnosed T2DM | Phenotypic classification feasibility | Not applicable (cluster analysis) | Standardization of core metabolic variables | Not applicable | Not applicable | The stability of the three classification scheme is better than that of the five classification scheme | Dependence on unconventional variables, lack of longitudinal validation |
| 40 | DKD | Cross sectional/prospective cohort | GTT study, T1D exchange registration | 836/8771 | T1DM adult | DKD risk | Continuous variable, logistic regression/cox | Age, onset age, gender, education, course of disease, BMI, waist hip ratio, blood pressure, blood lipids | OR=1.00 (GTT, P>0.05) | Linear (without RCS) | The correlation with IR was very weak (R=-0.25), much weaker than TyG (R=-0.64) | Lack of WHR data, adult T1DM only |
| 42 | CKD | Cross sectional study | CHARLS | 11,608 | Middle aged and elderly people over 45 years old | Serum creatinine/eGFR | Continuous variable, quartile | Gender, age, lifestyle, complications | Creatinine increased by 3.67 mg/dL (Q4 vs Q1) | Not reported | Not compared | Cross section, no longitudinal data |
| 43 | CKD | Prospective cohort | Wuhan Union Medical College Hospital physical examination crowd | 3,485 | Healthy subjects without CKD at baseline | Newly issued CKD within 3 years | Continuous variable, quartile, RCS | Age, gender, BMI, blood pressure, blood glucose, blood lipids, liver and kidney function, uric acid, etc | OR=4.24 (T3 vs T1) | Linear positive correlation (non linear P not reported) | AUC=0.857, Female/age<60/BMI ≥ 24 subgroup is stronger | CKD diagnosis is based on eGFR, only for Chinese |
| 44 | CKD | Prospective cohort | CHARLS | 6,117 | Middle aged and elderly people over 45 years old | New CKD risk | Cumulative mean (continuous, quartile, RCS) | Age, gender, smoking, drinking, education, chronic medical history | OR=1.45 (Q4 vs Q1) | Linear positive correlation (P-nonlinear=0.139) | Use cumulative average to reflect long-term exposure | CKD diagnosis is based on self-report |
| 45 | CKD | Cross sectional study | NHANES | 11,491 | Adults over 20 | CKD, CVD, Risk of cardiorenal syndrome (CRS) | Continuous variable, quartile, RCS | Age, gender, race, income, smoking, hypertension, diabetes, etc | CKD: OR=1.39 (Q4 vs Q1) | CKD: nonlinear (P-nonlinear<0.05) CVD/CRS: linear | The predictive power is weaker than TyG-WHtR and TyG- WC | Cross sectional design, CVD based on self-report |
| 46 | CKD | Cross sectional study | Zhuhai Wanshan community | 2,713 | Community residents aged 18-75 | CKD risk | Continuous variable, quartile | Age, gender, education, exercise, smoking, drinking, blood pressure, blood lipids, creatinine, uric acid, hs CRP, insulin | OR=1.65 (Q4 vs Q1, not significant after incomplete adjustment) | Linear (without RCS) | The predictive power was weaker than TyG-WHtR (AUC 0.648 vs 0.687) | Cross section, single center, South China community only |
| 47 | CKD | Retrospective cohort | NHANES | 3,089 | CKD patients | All cause and cardiovascular mortality | Continuous variable, quartile, RCS | Age, gender, race, education, income, smoking, hypertension, diabetes, blood lipids, UACR, eGFR | All cause death: HR=0.74 (Q4 vs Q1) | U-shaped nonlinearity (P<0.001) Inflection point: 299.31 | Better than TyG (AUC 0.574 vs 0.527) | Retrospective, single measurement, lack of etiological stratification |
| 48 | CKD | Prospective observation | Kaohsiung Medical University Hospital | 2,457 | Patients with stage 1-4 CKD | Adverse renal outcomes/all-cause death | Continuous variable, quartile | Age, gender, eGFR, urinary protein, cardiovascular disease, smoking, cancer, liver disease, hypertension, hemoglobin, BMI, HbA1c, CRP | Adverse renal outcome: HR=1.86 (Q1 vs Q4) | Inverse linear trend (TyG in U shape) | TyG index has the best independent predictive value | Single center, potential hybrid |
| 49 | CKD | Prospective cohort | CHARLS | 3,037 | Middle aged and elderly people over 45 years old | Risk of chronic comorbidity (MCD) | Continuous variable, quartile, RCS | Gender, age, marriage, education, BMI, smoking, drinking, sleep duration | HR=2.17 (Q4 vs Q1) | Nonlinear (p<0.0001) Inflection point: 171 | The predictive power was weaker than TyG (AUC 0.590 vs 0.610) | Self reported bias, only two time points |
| 50 | CKD | Cross sectional study | CHARLS | 8,177 | Middle aged and elderly people over 45 years old | 9 chronic disease risks | Continuous variable, RCS | Gender, age, place of residence, education, region, marriage, smoking, drinking, disability, diabetes, BMI | Hypertension: OR=1.05 (per unit increment) | Non linear with heart disease/digestive disease/hyperuricemia (inflection point 204.66) | TyG was the best predictor of dyslipidemia (AUC 0.809) | Cross section, lack of insulin data |
| 51 | RRT | Multicenter retrospective cohort | Four peritoneal dialysis centers in China | 1736 | Peritoneal dialysis (PD) patients | All cause mortality | Continuous variable, normalized Z-value, binary variable, RCS | White blood cells, hemoglobin, albumin, uric acid, kt/v, diabetes, cardiovascular disease, etc | HR=1.43 (high vs low TyG-BMI group) | Linear (P-nonlinear=0.716) | Less predictive than TyG index | Retrospective design, lack of inflammatory markers |
| 54 | CRMM | Prospective cohort | CHARLS | 7,848 | Middle aged and elderly people over 45 years old | Risk of cardiorenal metabolic comorbidity (CRMM) | Continuous variable, quartile, RCS | Age, gender, place of residence, marriage, education, smoking, drinking, exercise, hypertension, dyslipidemia, hyperuricemia, etc | HR=1.40 (Q4 vs Q1) | Linear (P-nonlinear>0.05) | No direct comparison of different derived indicators | Self reported bias, residual confounding |
| 55 | CRMM | Prospective cohort | UK Biobank | 327,692 | Adults without underlying cardiorenal metabolic diseases | Risk of occurrence and progression to multiple comorbidities | Continuous variable (per SD increment), RCS | Age, gender, race, education, smoking, drinking, HbA1c, LDL-C | Level I: HR=1.43 Level III: HR=1.97 (per SD increment) | Nonlinear (P-nonlinear<0.001) | The predictive power is weaker than TyG-WHtR and METS-IR | Selection bias, single baseline measurement |
| 56 | CRMM | Prospective cohort | UK Biobank | 349,974 | Adults without underlying cardiorenal metabolic diseases | Risk of occurrence and progression to multiple comorbidities | Continuous variable (per SD increment), RCS | Age, gender, race, education, smoking, drinking, history of hypertension, blood lipids | Level I: HR=1.32 Level III: HR=1.23 (per SD increment) | Nonlinear (P-nonlinear<0.001) | The strongest association with T2DM (HR=1.86) | Observational design, selection bias |
| 57 | CRMM | Prospective cohort | TLGS (Iran) | 5,845 | Adults over 30 | CRMM risk | Continuous variable (per SD increment), quintile, RCS | Age, gender, education, smoking, blood pressure, medication, blood lipids, blood glucose, etc | HR=2.57 (Q5 vs Q1) | Linear (P-nonlinear>0.05) | The predictive power is equivalent to the TyG index alone | Single center, urban population of Iran only |
| 58 | CKM | Prospective cohort | CHARLS | 765 | Patients with chronic liver disease (CLD) | Risk of CKM syndrome | Baseline value+trajectory clustering (K-means) | Age, gender, marriage, place of residence, education, smoking, drinking | HR=2.40 (high track group vs low group) | Linear trend (without RCS) | The risk of descent trajectory group is similar to that of low group (indicating the protective effect) | CLD diagnosis is based on self-report, only two time points |
| 59 | CKM | Retrospective cohort | NHANES | 6,383 | CKM 0-3 adults aged 30-79 | All cause and cardiovascular mortality | Continuous variable, quantile, smooth curve fitting | Age, gender, race, income, eGFR, UACR, uric acid, blood pressure, blood lipids, HbA1c, medication, complications | All cause death: HR=1.40 (T3 vs T1, P=0.342) | L-shaped nonlinearity (P<0.05) Inflection point: 169.05 | TyG-WHtR and TyG-WC have stronger predictive power | Observational, single measurement, selection bias |
| 60 | CKM | Retrospective cohort | NHANES | 10,622 | CKM 0-4 adults over 20 years old | All cause/cardiovascular/diabetes mortality | Continuous variable (TyG-BMI/10), quartile, smooth curve fitting | Age, gender, race, education, income, BUN, uric acid, creatinine, HDL, LDL, smoking, drinking, hypertension, diabetes | All-cause mortality: TyG HR=1.12 (1.01–1.24), Q4 vs Q1; DM mortality: TyG HR=1.72 (1.32–2.25); TyG-BMI and WTI not significant; TyG-WC/TyG-WHtR not significant for any mortality | U-shaped nonlinearity (P<0.001) Inflection point: 202.1 | TyG index is better in predicting all-cause and diabetic death | Retrospective, single measurement, USA only |
| 61 | CKM | Retrospective cohort | MIMIC-IV | 1,885 | Patients with CKM stage 4 (with CVD) in ICU | 180 day/1 year all-cause mortality | Continuous variable (per SD increment), quartile, RCS | Age, gender, BMI, marriage, hypertension, myocardial infarction, heart failure, stroke, AKI, laboratory indicators | 180 day death: HR=0.50 (Q4 vs Q1) | L-shaped linear negative correlation (P-nonlinear>0.05) | Better than BMI, TG, blood glucose alone | Single center, short-term follow-up, lack of eGFR, etc |
| 62 | CKM | Prospective cohort | CHARLS | 7,376 | CKM 0-3 adults over 45 years old | New CVD risk | Continuous variable (per 10 units), quartile, RCS | Age, gender, smoking, drinking, sleep, education, marriage, renal function, blood lipids, CRP, uric acid, hypertension, diabetes, depression | HR=1.80 (Q4 vs Q1) | Linear (P-nonlinear=0.355) | Not compared with other IR indicators | CVD is based on self-report, only for middle-aged and elderly people in China |
| 63 | CKM | Prospective cohort | CHARLS | 7,364 | CKM 0-3 adults over 45 years old | New CVD risk | Continuous variable (per SD increment), quartile, RCS | Age, gender, marriage, education, smoking, drinking, history of hypertension, history of diabetes, renal function, uric acid, eGFR, LDL | HR=1.70 (Q4 vs Q1) | Linear (P-nonlinear=0.446) | The predictive power was weaker than TyG-WC and TyG- WHtR (c-index 0.616 vs 0.621) | CVD is based on self-report, single center |
| 64 | CKM | Prospective cohort | Guangzhou Elderly Health Cohort | 463,545 | No CVD in CKM stage 0-3 over 65 years old | New CVD risk | Continuous variable, RCS | Age, gender, education, marriage, occupation, smoking, drinking, exercise, BMI, blood lipids, creatinine, WBC, medication | AUC=0.665 (TyG-BMI) | Non linearity (with other indicators) | TyG-WC is the best (AUC=0.763) | Short follow-up, self-reported outcome, lack of HOMA-IR |
| 65 | CKM | Prospective cohort | CHARLS | 5,782 | No CVD in CKM stage 0-3 over 45 years old | New CVD risk | Continuous variable (per SD increment), quartile, RCS | Age, gender, marriage, education, residence, smoking, drinking, hypertension, diabetes, dyslipidemia, creatinine, CRP | HR=1.50 (Q4 vs Q1) | Nonlinear (P-nonlinear<0.05) | TyG-WC is the best (c-index 0.643) | CVD self reporting, internal validation only |
| 66 | CKM | Prospective cohort | UK Biobank | 282,920 | CKM 0-3 adult without CVD | New onset CVD and mortality | Continuous, quartiles, RCS, Cox; NRI/IDI/AUC | Age, sex, ethnicity, TDI, education, income, employment, smoking, alcohol, PA, sleep, diet, SBP, DBP, HbA1c, TC, LDL-C | Overall CVD: TyG HR=1.30, TyG-BMI HR=1.49, TyG-WC HR=1.58, TyG-WHtR HR=1.58 (Q4 vs Q1, all p<0.001). All-cause mortality: TyG-BMI HR=1.11, TyG-WC HR=1.24, TyG-WHtR HR=1.18; CVD mortality: TyG-BMI HR=1.42, TyG-WC HR=1.51, TyG-WHtR HR=1.48 (all significant); TyG index not significant for mortality | TyG: Linear for overall CVD/CHD; nonlinear for stroke (inflection 8.474) and all-cause mortality (8.691). TyG-BMI/WC/WHtR: Nonlinear for overall CVD and CHD | Systematic comparison of TyG, TyG--WC,TyG-WHtR | Observational design, single measurement |
| 67 | CKM | Retrospective cohort | NHANES + CHARLS | 18158+5695 | CKM 0-3 adult | All cause and cardiovascular mortality | Continuous variable, quartile, RCS | Age, gender, race, education, income, diet, exercise, blood lipids, HbA1c, eGFR, UACR, complications, medication | All cause death: HR=2.07 (Q4 vs Q1) | Linear (P-nonlinear>0.05) | TyG-ABSI has the best predictive power (AUC 0.688-0.708) | Observability, single measurement, mediation analysis based on cross section |
| 70 | RRT | Single center retrospective cohort | Tianjin First Central Hospital | 865 | PD patients | All cause and cardiovascular mortality | Continuous variable, quartile, RCS | Gender, age, diabetes, blood pressure, hemoglobin, albumin, residual renal function, etc | All cause death: HR=1.84 (high group vs middle group) | U-shaped/J-shaped nonlinearity (P-nonlinear<0.05) | Not compared with other IR indicators | Single center, single baseline measurement |
| 71 | RRT | Single center retrospective cohort | The First Affiliated Hospital of Sun Yat sen University | 2,335 | PD patients | All cause and cardiovascular mortality | Continuous variable (per SD increment), quartile | Age, gender, systolic blood pressure, cardiovascular history, hemoglobin, albumin, LDL-C, urine volume, lipid-lowering drugs | Cardiovascular death: HR=1.51 (Q4 vs Q1) | Linear trend (without RCS) | Not compared with other IR indicators | Single center, lack of blood glucose prescription information |
| 73 | RRT | Cross sectional study | Hemodialysis center of Yangzhong people's Hospital | 219 | Maintenance hemodialysis (MHD) patients | Severity of coronary artery calcification (CAC) | Continuous variable, RCS, WQS | Age, gender, smoking, drinking, diabetes, hypertension, dialysis duration, blood pressure, blood glucose, HDL, hemoglobin, albumin, CRP | OR=1.011 (per unit increment) | Linear (P-nonlinear=0.412) | BMI component contributed the most (weight 0.566) | Single center, small sample, cross section |
| 74 | RRT | Multicenter retrospective cross-sectional study | Affiliated Hospital of Wenzhou Medical University | 297 | MHD patients | Risk of abdominal aortic calcification (AAC) | Continuous variable, binary variable, RCS | Age, gender, dialysis age, smoking, diabetes, hypertension, cardiovascular disease, iPTH, Kt/V, statins | OR=3.38 (high group vs low group) | Both linearity and nonlinearity were significant (P=0.001/0.014) | PHR is more stable | Small sample, cross section, AUC confidence interval width |
| 75 | RRT | Single center prospective cohort | Beijing Haidian Hospital | 249 | ESRD patients undergoing first AVF | AVF mature failure risk | Continuous variable, RCS | Age, gender, BMI, diabetes, hypertension, arteriosclerosis, laboratory indicators | HR=2.68 (per unit increment) | Linear (P-nonlinear=0.629) | Predictive power was weaker than SIRI (AUC 0.592 vs 0.668) | Single center, small sample, follow-up for 3 months |
| 76 | RRT | Retrospective cohort | First Affiliated Hospital of Wenzhou Medical University | 191 | Renal transplant recipients | Risk of post transplant diabetes mellitus (PTDM) | Continuous variable, tertiles, ROC analysis with Youden index for optimal cutoffs | age, sex,smoking, drinking, obesity, hypertension, hepatitis C,CNI type, TC, LDL‑C, ALB, ALT, eGFR, Hb | OR > 3.0 (highest tertile vs lowest, FK506 subgroup) | Positive linear trend | TyG-BMI AUC = 0.775, superior to TyG, TG/HDL-C, non-HDL-C/HDL-C (P < 0.001) | Retrospective, small sample, single-region |

The table contains numerous medical abbreviations, such as AAC (Abdominal Aortic Calcification), AKI (Acute Kidney Injury), ALB (Albumin), ALT (Alanine Aminotransferase), ApoB (Apolipoprotein B), AUC (Area Under the Curve), AVF (Arteriovenous Fistula), BMI (Body Mass Index), BUN (Blood Urea Nitrogen), CABG (Coronary Artery Bypass Grafting), CAC (Coronary Artery Calcification), CHARLS (China Health and Retirement Longitudinal Study), CHD (Coronary Heart Disease), CIN (Contrast‑Induced Nephropathy), CKD (Chronic Kidney Disease), CKM (Cardiovascular‑Kidney‑Metabolic Syndrome), CLD (Chronic Liver Disease), CNI (Calcineurin Inhibitor), CRMM (Cardio‑Renal‑Metabolic Multimorbidity), CRP (C‑reactive Protein), CRS (Cardiorenal Syndrome), C‑statistic (Concordance statistic), CVD (Cardiovascular Disease), DBP (Diastolic Blood Pressure), DKD (Diabetic Kidney Disease), DN (Diabetic Nephropathy), DPN (Diabetic Peripheral Neuropathy), DR (Diabetic Retinopathy), eGFR (estimated Glomerular Filtration Rate), eICU‑CRD (eICU Collaborative Research Database), ESRD (End‑Stage Renal Disease), FCP (Fasting C‑peptide), FT4 (Free Thyroxine), GTT (Glucose Tolerance Test), Hb (Hemoglobin), HbA1c (Hemoglobin A1c), HDL‑C (High‑Density Lipoprotein Cholesterol), HOMA‑IR (Homeostatic Model Assessment for Insulin Resistance), HR (Hazard Ratio), hs‑CRP (high‑sensitivity C‑reactive protein), ICU (Intensive Care Unit), IDI (Integrated Discrimination Improvement), iPTH (intact Parathyroid Hormone), IR (Insulin Resistance), Kt/V (urea clearance index), LDL‑C (Low‑Density Lipoprotein Cholesterol), MCD (Multiple Chronic Diseases), METS‑IR (Metabolic Score for Insulin Resistance), MHD (Maintenance Hemodialysis), MIMIC‑IV (Medical Information Mart for Intensive Care IV), NAFLD (Non‑Alcoholic Fatty Liver Disease), NHANES (National Health and Nutrition Examination Survey), NRI (Net Reclassification Improvement), NT‑proBNP (N‑terminal pro‑brain natriuretic peptide), OR (Odds Ratio), PA (Physical Activity), PCI (Percutaneous Coronary Intervention), PCT (Procalcitonin), PD (Peritoneal Dialysis), PHR (Platelet‑to‑High‑density lipoprotein cholesterol Ratio), PTDM (Post‑Transplant Diabetes Mellitus), RCS (Restricted Cubic Spline), ROC (Receiver Operating Characteristic), RRT (Renal Replacement Therapy), SAPS II (Simplified Acute Physiology Score II), SBP (Systolic Blood Pressure), SD (Standard Deviation), SIRI (Systemic Inflammation Response Index), SIRS (Systemic Inflammatory Response Syndrome), SOFA (Sequential Organ Failure Assessment), STEMI (ST‑Segment Elevation Myocardial Infarction), T1D (Type 1 Diabetes), T1DM (Type 1 Diabetes Mellitus), T2DM (Type 2 Diabetes Mellitus), TC (Total Cholesterol), TDI (Townsend Deprivation Index), TG (Triglyceride), TLGS (Tehran Lipid and Glucose Study), TyG (Triglyceride‑Glucose Index), TyG‑ABSI (Triglyceride‑Glucose Index‑A Body Shape Index), TyG‑BMI (Triglyceride‑Glucose Index‑Body Mass Index), TyG‑WC (Triglyceride‑Glucose Index‑Waist Circumference), TyG‑WHtR (Triglyceride‑Glucose Index‑Waist‑to‑Height Ratio), UA (Uric Acid), UACR (Urine Albumin‑to‑Creatinine Ratio), UK Biobank (UK Biobank), WBC (White Blood Cell count), WQS (Weighted Quantile Sum regression), and WTI (Waist Triglyceride Index).
